# Phenotypic Subacute Toxicity Assessment of Intranasally Administered Larixyl Acetate: Implications for Potential Airway Applications

**DOI:** 10.3390/jox16030100

**Published:** 2026-06-01

**Authors:** Zaina Kalaji, Ibrahim Hachim, Marwa Almazrouei, Hanaa Habbal, Vidya Bijosh Mohan, Mohammad G. Mohammad, Rifat Hamoudi, Rabih Halwani

**Affiliations:** 1 Research Institute of Medical and Health Sciences, College of Medicine, University of Sharjah, Sharjah P.O. Box 27272, United Arab Emirates; ihachim@sharjah.ac.ae (I.H.); marwa.almazrouei@sharjah.ac.ae (M.A.); hhabbal@sharjah.ac.ae (H.H.); vmohan@sharjah.ac.ae (V.B.M.); mmohd@sharjah.ac.ae (M.G.M.); rhamoudi@sharjah.ac.ae (R.H.); rhalwani@sharjah.ac.ae (R.H.); 2 Clinical Sciences Department, College of Medicine, University of Sharjah, Sharjah P.O. Box 27272, United Arab Emirates; 3 Department of Medical Laboratory Sciences, College of Health Sciences, University of Sharjah, Sharjah P.O. Box 27272, United Arab Emirates; 4 Division of Surgery and Interventional Science, University College London, London NW3 2PF, UK; 5 Biomedically Informed Artificial Intelligence Laboratory (BIMAILab), University of Sharjah, Sharjah P.O. Box 27272, United Arab Emirates; 6 Center of Excellence for Precision Medicine, University of Sharjah, Sharjah P.O. Box 27272, United Arab Emirates; 7 ASPIRE Precision Medicine Research Institute Abu Dhabi, University of Sharjah, Sharjah P.O. Box 27272, United Arab Emirates; 8 Health and Wellbeing, NEOM, Tabuk P.O. Box 11149, Saudi Arabia; 9 King Saud University Celiac Disease Research Chair, Department of Pediatrics, College of Medicine, King Saud University, Riyadh P.O. Box 2454, Saudi Arabia; 10 Prince Fahad Bin Sultan Chair for Biomedical Research, University of Tabuk, Tabuk P.O. Box 11149, Saudi Arabia; 11 College of Medicine, Alfaisal University, Riyadh P.O. Box 2454, Saudi Arabia

**Keywords:** Larixyl acetate, subacute toxicity, intranasal delivery, OECD Test Guideline 412

## Abstract

Larixyl acetate, a primary component of Larch turpentine, is a naturally occurring compound with a broad spectrum of medicinal properties, including anti-inflammatory effects. It is a potent and selective inhibitor of TRPC6, a widely expressed Ca^2+^ channel that is involved in many respiratory diseases. Despite its demonstrated efficacy, it lacks a well-defined preclinical and phenotypic safety profile, which limits its therapeutic potential and implementation. In this study, female BALB/c mice were used to assess the toxicity of intranasally administered Larixyl acetate through a subacute model based on OECD Test Guideline 412, followed by a detailed analysis of physical, blood, biochemical, and tissue changes at the administration sites and beyond. Within the study’s 30-day timeframe, our results show no statistically significant differences (*p* > 0.05) in any of the examined toxicity parameters between the controls or three treatment groups (0.5, 1, and 2 mg/kg). While no pharmacokinetic data were obtained to confirm local or systemic exposure of Larixyl acetate, these findings are crucial for establishing a solid foundation for future therapeutic endeavors, especially in the context of TRPC6-driven respiratory diseases.

## 1. Introduction

Over the past decade, the pharmaceutical industry has seen a significant shift toward natural and plant-based treatments for many inflammatory and malignant conditions, due to their effectiveness and limited toxicity or side effects [1,2]. Especially when compared with contemporary compounds such as corticosteroids and non-steroidal anti-inflammatory drugs (NSAIDs) [3,4]. One such example is Larixyl acetate, a labdane-type diterpene derived from several Larch tree species [5,6], which exhibits a range of therapeutic properties, including anti-inflammatory, antioxidant, antitumor, antifungal, and antibacterial effects [5,7,8]. For instance, in vitro evidence underscores Larixyl acetate’s relatively good capacity to limit leukotriene B4 (LTB_4_) production, an important mediator of inflammatory responses in allergic airway diseases. Additionally, in vivo murine evidence further emphasizes its cerebrovascular, cardiovascular, and neuroprotective roles against traumatic brain injury (TBI)-mediated endothelial dysfunction, chronic hypertension-induced heart failure, and neuropathic pain, respectively [9,10,11]. This diverse profile of therapeutic modalities marks Larixyl acetate as a promising agent worthy of further characterization for other therapeutic applications.

A defining study by Urban et al. in 2016 was the first to document Larixyl acetate’s molecular targets and mode of action, by suppressing calcium (Ca^2+^) signaling through inhibition of transient receptor potential canonical channel 6 (TRPC6) activity [6,12]. In the context of pulmonary and airway diseases, the pathogenic overexpression and activation of TRPC6 were attributed to inflammatory responses driving nasal polyposis [13], lung edema, idiopathic pulmonary hypertension, and pulmonary fibrosis [6,14,15]. These pathological artifacts are partly due to the ubiquitous expression of TRPC6 in bronchial epithelial cells, lung macrophages, lung myofibroblasts, and bronchial smooth muscle cells [6,15,16]. Accordingly, Larixyl acetate was employed in treating said airway disorders, where it effectively suppressed oxidative stress-induced Ca^2+^ mobilization in human bronchial epithelial cells, which ultimately reduced airway inflammation [16].

More importantly, Larixyl acetate showed a favorable profile when tested in vitro and ex vivo, with minimal cytotoxicity and reversible inhibition upon cessation of administration [6]. Its favorable electrophysiological, toxicological, and biological characteristics make it suitable for disease-model treatment; however, the lack of prior in vivo acute or chronic toxicity assessments limits the translation and clinical interpretation of these findings. Additionally, most of these studies used intraperitoneal, cutaneous, or intrathecal routes, with little to no data on the commonly used intranasal route, which is most prominently elected in models of airway diseases where TRPC6 is pathophysiologically involved [9,10,11]. Accordingly, it is of paramount importance to properly test for potential short- and long-term toxic effects of Larixyl acetate through other routes of administration.

Therefore, given the clear and promising therapeutic potential of Larixyl acetate and the lack of in vivo toxicity data from mouse models, we aimed to characterize its phenotypic subacute toxicological profile using BALB/c mice, which are ideal for immunological assessments in airway diseases. Consequently, the mice were used to evaluate the phenotypic toxicity profile of various intranasally administered doses and to determine the most effective concentration with a non-significant toxicity profile for future therapeutic research. Intranasal administration was specifically chosen because it represents the clinically relevant and intended route of delivery for future therapeutic use of Larixyl acetate in upper-airway inflammatory diseases such as allergic rhinitis and chronic rhinosinusitis with nasal polyps (CRSwNP), conditions in which TRPC6 has been implicated. In humans, these diseases are routinely managed with intranasal sprays; therefore, testing the compound via the intranasal route in mice provides data that are directly translatable to potential clinical application. This route also enables localized delivery to the target nasal and upper-airway mucosa while minimizing systemic exposure. Thus, our study aims to lay a foundation for future research on Larixyl acetate for treating TRPC6-related respiratory conditions by leveraging its medicinal benefits.

## 2. Materials and Methods

### 2.1. Animals and Ethics Statement

The experiments on mice were approved by the Animal Care and Use Committee at the University of Sharjah (Approval No. ACUC-20-02-11-01) and adhered to the country’s regulations for the care and use of laboratory animals. This research involved female wild-type BALB/c mice aged 6–7 weeks, obtained from the University of Sharjah’s animal facility. All mice meeting the animal facility’s health screening criteria were included in the study; no animals were excluded from any experimental group or data analysis. The animals were housed in sterile, pathogen- and Ovalbumin (OVA)-free cages with individual ventilation (TECNIPLAST, Tecniplast Group, Buguggiate, Italy). The mice were exposed to a 12 h light–dark cycle and maintained at appropriate room temperatures (20–26 °C) and relative humidity (35–70%). They were also provided with a sterile maintenance diet (Altromin 1324 TPF, Altromin Spezialfutter GmbH & Co. KG, Lage, Germany) and given access to sterile distilled water. The enclosures contained autoclaved aspen bedding free of dust (ABEDD). All mice were acclimated for 3 days prior to starting the experiments.

### 2.2. Experimental Design of the Subacute Toxicity Study

Our experimental model was conducted following the general principles and recommended endpoints of the Organization for Economic Co-operation and Development (OECD) Test Guideline 412 for subacute inhalation toxicity [17], adapted for intranasal instillation as a practical surrogate for local upper-airway delivery rather than whole-body or nose-only aerosol exposure. The 30-day duration was selected as it represented a standard period for sub-acute toxicity assessments, allowing evaluation of cumulative effects from repeated daily intranasal dosing while remaining practical for an initial phenotypic safety screen before longer-term studies. Female mice weighing 20 ± 0.14 g were randomly assigned to four groups to assess the safety profile of three different doses of Larixyl acetate (Cat. No.: HY-101795, MedChemExpress, Monmouth Junction, NJ, USA). Twenty-four mice were divided into the four treatment groups: Control (PBS-treated), Larixyl acetate (LA)-treated (0.5 mg/kg), Larixyl acetate-treated (1 mg/kg), and Larixyl acetate-treated (2 mg/kg). The doses were selected on the basis of published therapeutic intraperitoneal doses of Larixyl acetate that have been shown to be effective with no notable toxicity in mice models of traumatic brain injury (5 mg/kg) [9], pressure overload-induced heart failure (5 mg/kg/day) [10], and ozone-induced airway inflammation [16], as well as the related TRPC6 inhibitor SH045 (20 mg/kg single dose) in pharmacokinetic and renal fibrosis studies [18]. These intraperitoneal doses produced plasma levels well above the low-nanomolar IC_50_ for TRPC6 inhibition while remaining safe. Given that intranasal administration may result in rapid systemic absorption via the nose-to-heart pathway [19], we deliberately chose intranasal doses that are 2.5- to 10-fold lower than the established safe and effective intraperitoneal doses to provide a conservative safety margin in this foundational OECD-guided toxicity assessment. The highest dose (2 mg/kg) is also within the range used for other intranasally administered Ca^2+^-channel modulators in murine airway models [20].

Larixyl acetate is lipophilic; therefore, it was solubilized in DMSO and prepared daily as a fresh 20 µL suspension in a mixture consisting of Larixyl acetate in DMSO (2%) (Sigma-Aldrich, St. Louis, MO, USA), PEG 400 (5%) (Sigma-Aldrich, St. Louis, MO, USA), Tween-80 (0.5%) (Sigma-Aldrich, St. Louis, MO, USA), and PBS (92.5%) (Sigma-Aldrich, St. Louis, MO, USA), added to the final formulation in that order by vortexing and brief sonication to ensure homogeneity before administration. The PEG 400 and Tween-80 were essential components to ensure encapsulation, homogeneous suspension, and solubilization of the lipophilic compound in PBS. The low concentrations of DMSO (2%), PEG 400 (5%), and Tween-80 (0.5%) were carefully selected based on previous studies demonstrating that these excipients, at similar or higher levels, are well tolerated by the nasal mucosa, provide effective solubilization of lipophilic compounds, cause minimal irritation, and do not significantly stimulate mucin secretion or impair mucociliary clearance [20,21,22,23,24]. The 20 µL intranasal volume (≈10 µL per nostril) is standard for anesthetized mice in respiratory delivery studies and does not cause irritation or aspiration. All doses were administered daily and pipetted into each nostril via the intranasal route after sedation with isoflurane throughout the 30-day experimental period.

**Figure 1:**
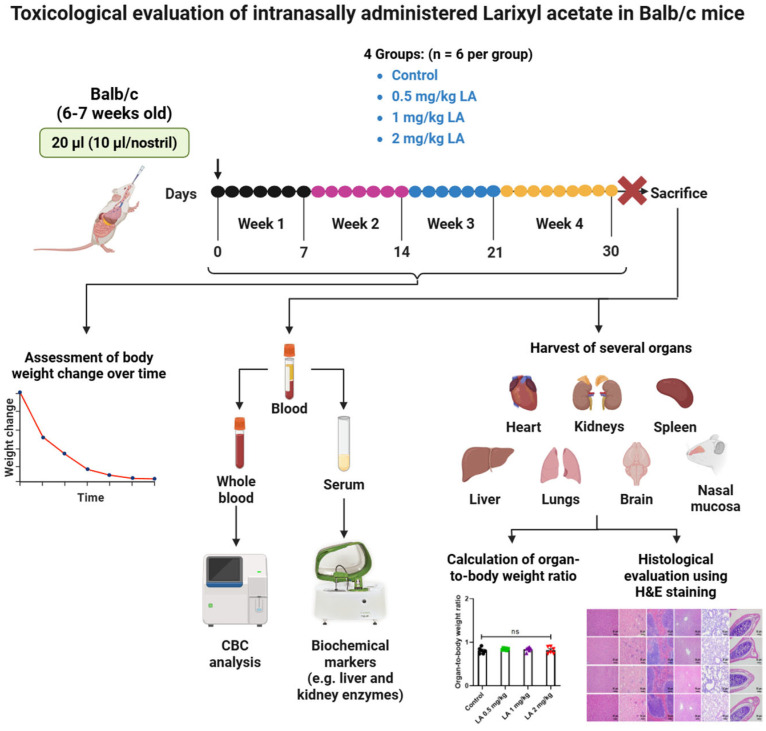
A schematic illustration showing the experimental setup, doses, and timeline for the sub-acute toxicity evaluation of intranasally administered Larixyl acetate (LA) (*n* = 6 mice/group). The schematic illustration was generated via Biorender.com.

Each group consisted of six mice (*n* = 6), with the sample size selected in accordance with OECD Test Guideline 412 recommendations for subacute inhalation toxicity studies and with previous intranasal toxicology investigations in mice. Mice were randomly assigned to the four treatment groups (*n* = 6 per group) using a computer-generated random number sequence (Microsoft Excel). To minimize potential cage effects and environmental bias, animals were stratified by body weight and then randomly allocated to treatment groups using blocked randomization (block size = 4). After the assignment, mice were housed in cages according to their treatment group (6 mice per cage), with cages placed on the same rack level and rotated weekly to ensure equal environmental exposure across groups. Daily animal welfare was assessed using a standardized scoring system that evaluated body condition, activity level, grooming, respiratory signs, and signs of distress, in accordance with institutional animal care guidelines. No animals displayed any transient or persistent clinical signs during the daily welfare assessments. No animals were excluded from the study, and there were no protocol deviations or attrition during the 30-day period. All dosing, clinical observations, body weight measurements, and data collection (except histopathological evaluation) were performed by the same trained personnel. The histopathologist was independently blinded to treatment allocation. A full schematic illustration of the experimental and study design is seen in (Figure 1).

### 2.3. Body Weight Changes over Time

Throughout the model, mice were weighed every 3 days. Changes in body weight were documented in grams (g). The values were later plotted over time to examine significant differences in body weight between groups.

### 2.4. Organ-to-Body Weight Index

At the end of the study, several organs were harvested and weighed, including the heart, kidneys, liver, spleen, lungs, and brain. Organ-to-body weight ratios were calculated using this formula:

$$Organ\text{-}to\text{-}body\;weight\;index=\;\frac{Organ\;weight\;(g)\times 100}{body\;weight\;(g)}$$

Ratios were plotted, and further analysis was performed to assess changes in overall organ size and mass.

### 2.5. Hematological Analysis

At the end of the study, 250 µL of mouse blood was collected into EDTA-coated tubes to prevent clotting. The blood samples were obtained via cardiac puncture after mice were anesthetized with intraperitoneal injections of ketamine (114.5 mg/kg) and xylazine (6.9 mg/kg). The collected blood was used for subsequent hematological assessments. A comprehensive blood count (CBC) was performed using the DxH 500 Hematology Analyzer (B40601, Beckman Coulter, Lane Cove West, NSW, USA) to measure various parameters of red blood cells (RBCs) and white blood cells (WBCs), including hemoglobin, total RBC count, mean corpuscular hemoglobin (MCH), mean corpuscular hemoglobin concentration (MCHC), platelet count, mean platelet volume (MPV), neutrophils, lymphocytes, monocytes, and eosinophils. The data were analyzed and compared.

### 2.6. Biochemical Analysis

Another 200–250 µL of whole blood was obtained on the day of sacrifice, followed by centrifugation at 720× *g* for 10 min at 4 °C. The serum was isolated for biochemical testing of the following liver and kidney enzymes: alkaline phosphatase (ALP), Aspartate aminotransferase (AST), Alanine aminotransferase (ALT), total bilirubin (TBIL), uric acid, and creatinine. Levels of these enzymes were measured using the automated clinical chemistry analyzer (Adaltis Pchem1, Rome, Italy). Values were plotted to identify any abnormalities in enzymatic levels among the groups.

### 2.7. Histopathological Assessment

Multiple organs, including the heart, kidneys, lungs, spleen, and liver, were preserved in 10% formalin for a duration of 24 h. Mouse skulls were fixed and subsequently decalcified using 5% HCL and 10% EDTA decalcifying solution (Ref no. 2117, GBL Microscopy, Ankara, Turkey) over a period of three days. Subsequently, a surgical blade was utilized to produce coronal sections at the nasal vestibule. All organs and sections underwent processing and embedding in paraffin. Sections with a thickness of 5 μm were prepared using a rotary microtome (SLEE Medical GmbH, Mainz, Germany). These sections were then stained with hematoxylin and eosin (H&E) (Cat No. PK10031, Proteintech, Rosemont, IL, USA). Histopathological evaluations were conducted by a board-certified histopathologist blinded to treatment groups. Semi-quantitative scoring of lesions followed the International Harmonization of Nomenclature and Diagnostic Criteria for Lesions in Rats and Mice (INHAND) guidelines [19]. A 0–4 scale was used with the following operational definitions: 0 = within normal limits (tissue considered to be normal under the conditions of the study and considering the age, sex, and strain of the animal concerned); 1 = minimal (the amount of change present barely exceeds that which is considered to be within normal limits); 2 = slight (the lesion is easily identified but of limited severity); 3 = moderate (the lesion is prominent, but there is significant potential for increased severity); 4 = severe (the degree of change is as complete as possible and occupies the majority of the organ). Five representative high-power fields (×400) were evaluated per section and averaged. For the nasal mucosa, infiltrating eosinophils and polypoid lesions were enumerated and averaged across five fields per sample. Organ-specific criteria (e.g., alveolar septal congestion, epithelial integrity, and inflammatory infiltrates) were applied as detailed in the INHAND respiratory and other organ-system guidelines. No additional quantitative morphometric analyses (e.g., nasal mucosa thickness or goblet cell counts) were performed because the observed changes were minimal and showed no clear treatment-related pattern.

### 2.8. Statistical Analysis

The GraphPad Prism software (version 8.0; GraphPad Software, La Jolla, CA, USA) was used to create figures and perform statistical analyses. Normality and homogeneity of variance were assessed prior to analysis, and all datasets met the assumptions required for parametric testing. In this study, data are shown as means ± SEM. For comparisons, either a One-Way analysis of variance (ANOVA) or a two-way ANOVA was conducted as appropriate, followed by Bonferroni post hoc tests for multiple comparisons. To complement the null-hypothesis testing and quantify the magnitude of treatment effects, overall effect sizes ($R^{2}$ values) and group-specific 95% confidence intervals (CIs) were calculated for the evaluated parameters. All statistical tests were two-tailed, and a *p*-value of less than 0.05 (*p* < 0.05) was considered statistically significant. *p*-values greater than 0.05 (*p* > 0.05) indicated no significant differences across the parameters.

## 3. Results

### 3.1. Intranasal Administration of TRPC6 Inhibitor, Larixyl Acetate, Does Not Alter Body Weight or Organ-to-Body Weight Ratios

**Figure 2:**
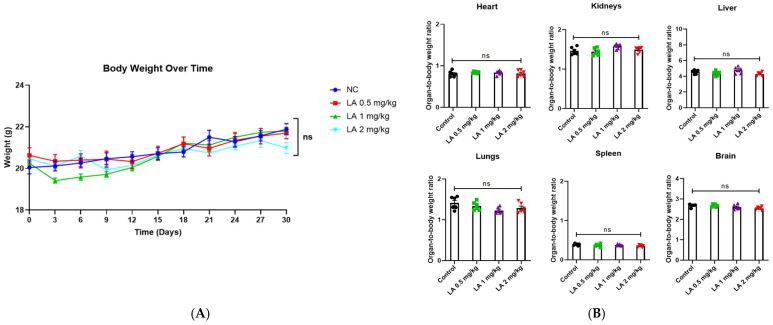
Intranasal administration of TRPC6 inhibitor, Larixyl acetate, does not alter body weight or organ-to-body weight ratios. (**A**) Body weight change in female BALB/c mice over the span of one month. (**B**) Organ-to-body weight ratios were assessed for various organs, revealing no observed statistical differences (*p* > 0.05) across all treatment groups at the end of the study. Statistical analyses were done using Two-way ANOVA or One-way ANOVA, followed by Bonferroni post hoc tests for multiple comparisons. Data are presented as mean ± SEM (*n* = 6 per group). Individual animal data points are shown as circles, squares, or triangles. (ns) indicates no statistically significant difference (*p* > 0.05).

Conducting this study was imperative, as in vivo toxicity evaluations for this compound were lacking in the literature, particularly for intranasal administration. Over the course of the model, we consistently measured changes in the mice’s body weights, and the results showed no statistically significant (*p* > 0.05) differences in body weight (Figure 2A), especially across the different dose groups.

Moreover, upon sacrificing the mice, the weights of several key organs were measured to assess the effects of drug-induced hypertrophy or shrinkage, including the heart, kidneys, liver, lungs, spleen, and brain. The organ-to-body weight ratios were subsequently calculated using the aforementioned formula. Accordingly, no statistically significant differences (*p* > 0.05) were observed in ratios between the three doses and the controls (Figure 2B), indicating the relative safety of Larixyl acetate on mouse growth rates under the tested conditions and study’s timeframe, with no drug-induced organ damage or size changes noted afterwards. The raw data for the ratios are presented as mean value ± SEM, complemented by group-specific 95% confidence intervals (CIs), global one-way ANOVA *p*-values, and overall effect sizes ($R^{2}$) as seen in (Table A1).

### 3.2. Intranasal Administration of TRPC6 Inhibitor, Larixyl Acetate, Is Associated with Minimal Hematological Alterations

**Figure 3:**
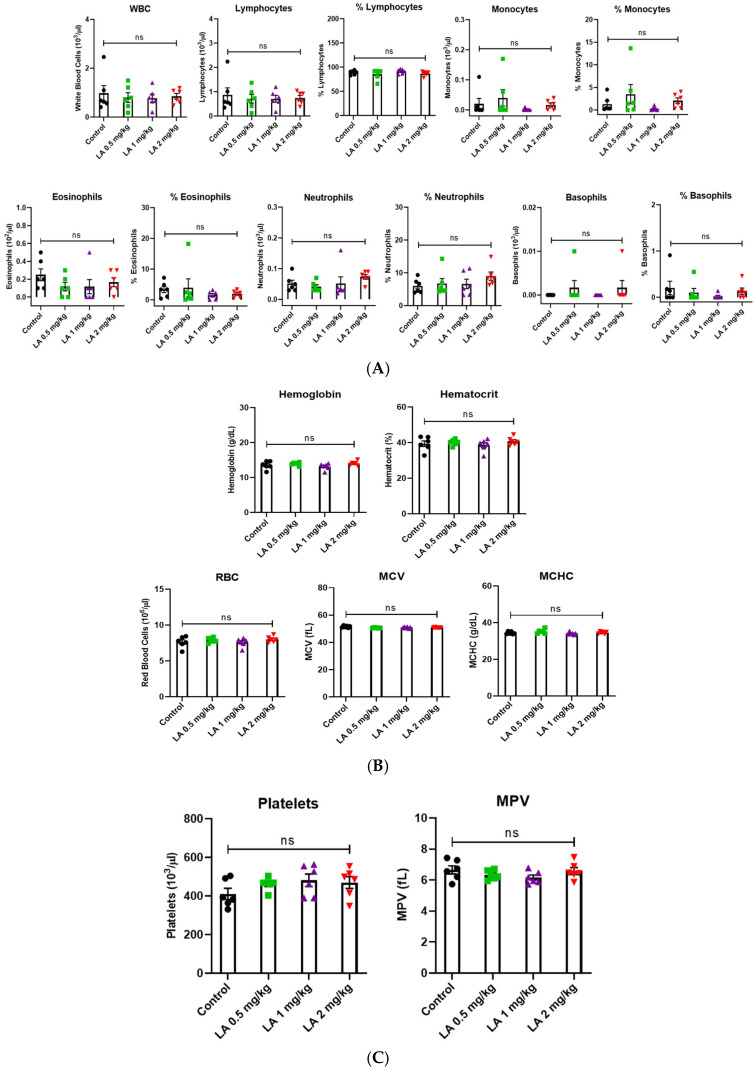
Intranasal administration of the TRPC6 inhibitor Larixyl acetate is associated with minimal hematological alterations. (**A**) Crude counts and percentages of WBCs, lymphocytes, monocytes, eosinophils, neutrophils, and basophils were plotted, showing no statistically significant (*p* > 0.05) differences. (**B**) Equally, counts of RBCs, hemoglobin, hematocrit, MCH, and MCHC showed no statistically significant (*p* > 0.05) differences, with relative parameters like (**C**) platelets and MPV reflecting a similar outcome. Statistical analyses were done using One-way ANOVA, followed by Bonferroni post hoc tests for multiple comparisons. Data are presented as mean ± SEM (*n* = 6 per group). Individual animal data points are shown as circles, squares, or triangles. (ns) indicates no statistically significant difference (*p* > 0.05).

To study the potential effects of Larixyl acetate on systemic hematological values that may reflect changes in immune function or clotting indices, a comprehensive CBC was performed to assess signs of anemia, leukocytosis, leukocytopenia, changes in leukocyte subpopulations, or clotting abnormalities. The crude values and percentages were plotted and analyzed, reflecting statistically negligible variations (*p* > 0.05) in WBCs, lymphocytes, monocytes, eosinophils, neutrophils, and basophils across all treatment groups (Figure 3A). These findings negate any prominent inflammatory or immunosuppressive actions for Larixyl acetate within the study’s timeframe.

Furthermore, red blood cell (RBC) counts and indices reflected statistically insignificant differences (*p* > 0.05), as evidenced by consistent hemoglobin, hematocrit, mean corpuscular volume (MCV), and mean corpuscular hemoglobin concentration (MCHC) measurements (Figure 3B). Likewise, platelet counts and mean platelet volume (MPV) levels were not statistically significant (*p* > 0.05) (Figure 3C). These findings indicate the relative absence of anemia or thromboembolic events across the tested doses, thereby further corroborating the safety profile of the pharmaceutical agent within the studied timeframe. A comprehensive overview of hematological parameters as presented by the raw mean value ± SEM, complemented by group-specific 95% confidence intervals (CIs), global one-way ANOVA *p*-values, and overall effect sizes ($R^{2}$) as seen in (Table A2).

### 3.3. Intranasal Administration of TRPC6 Inhibitor, Larixyl Acetate, Is Associated with No Overt Hepatic or Renal Toxicity

To investigate the potential effects of Larixyl acetate on hepatic and renal functions, the levels of several key enzymes released in response to drug-induced tissue damage or cellular necrosis were evaluated. Since both organs are primary sites of drug metabolism and excretion, respectively, any internal cellular injury is promptly reflected in the circulation and can be easily measured. Accordingly, the levels of multiple enzymes associated with tissue damage, indicative of hepatotoxicity—such as alkaline phosphatase (ALP), aspartate aminotransferase (AST), alanine aminotransferase (ALT), and total bilirubin (TBIL)—were assessed. Additionally, markers of nephrotoxicity, including uric acid and creatinine, were also examined.

**Figure 4:**
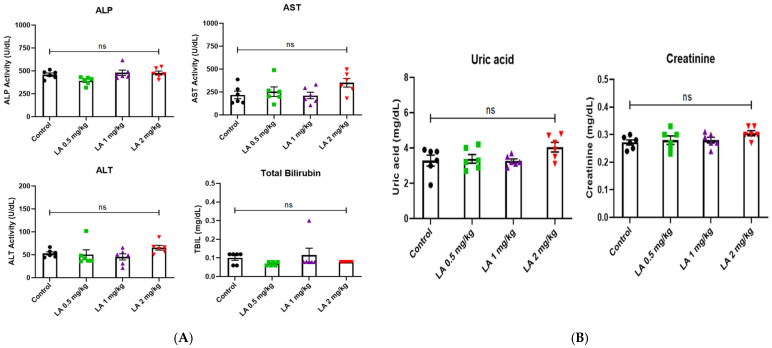
Intranasal administration of TRPC6 inhibitor, Larixyl acetate, is associated with no overt hepatic or renal toxicity. (**A**) Levels of hepatotoxicity-associated enzymes, including ALP, AST, ALT, and TBIL, show no statistically significant (*p* > 0.05) change or increase. (**B**) Likewise, levels of nephrotoxicity-associated enzymes, including uric acid and creatinine, remained within a table range (*p* > 0.05). Statistical analyses were done using One-way ANOVA, followed by Bonferroni post hoc tests for multiple comparisons. Data are presented as mean ± SEM (*n* = 6 per group). Individual animal data points are shown as circles, squares, or triangles. (ns) indicates no statistically significant difference (*p* > 0.05).

Our findings at the end of the study’s 30-day timeframe reflect no statistically significant (*p* > 0.05) alterations in hepatic (Figure 4A) or renal (Figure 4B) enzymatic levels in response to any of the tested doses. This underscores another facet of Larixyl acetate’s safety profile under the examined conditions, as indicated by the absence of damage-associated biochemical markers in the liver or kidneys. Detailed data regarding these biochemical parameters are provided as mean value ± SEM, complemented by group-specific 95% confidence intervals (CIs), global one-way ANOVA *p*-values, and overall effect sizes ($R^{2}$) as seen in (Table A3).

### 3.4. Intranasal Administration of TRPC6 Inhibitor, Larixyl Acetate, Does Not Induce Significant Histopathological Alterations

**Figure 5:**
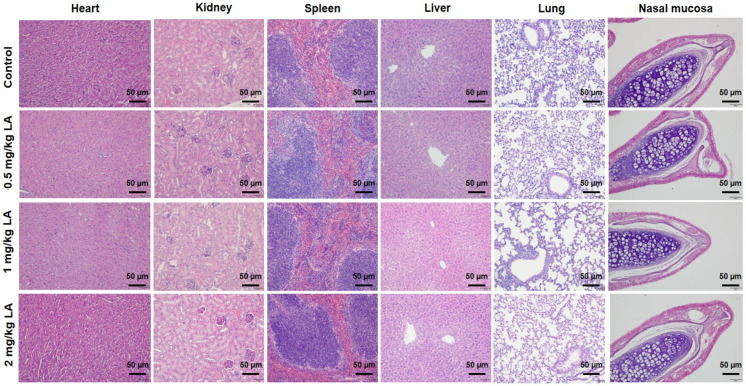
Intranasal administration of the TRPC6 inhibitor Larixyl acetate does not induce significant histopathological alterations. This figure displays representative sections of heart, kidneys, spleen, liver, lungs, and nasal mucosa stained with hematoxylin and eosin (H&E), collected from different mice (*n* = 6) across all control and treated groups. Original magnification ×20; scale bar = 50 μm.

To determine whether the intranasal administration of Larixyl acetate is associated with localized modifications in the nasal cavity’s architecture and/or systemic effects on vital organs, a histological analysis of the nasal mucosa and other organs was conducted. This definitive step is essential for validating the structural integrity of the affected organs and targeted sites. Consequently, several organs were harvested, including the heart, kidneys, spleen, liver, lungs, and the skull, which houses the nasal mucosa (Figure 5). These organs underwent further histopathological examination and were scored for indications of damage in relevant structural regions [25].

Overall, the results of this evaluation indicated no significant histological alterations between the control and treated groups. Evidently, minimal structural alterations were observed in cardiac tissue among mice from each treatment group, with all exhibiting normal cardiac muscle architecture, no inflammatory cell infiltration, and no necrosis. Furthermore, all mice’s kidneys showed no evidence of nephrotoxicity, maintaining normal renal cortex and glomerular tufts. Regarding the spleen, no aberrant artifacts were detected in the white or red pulp across all specimens. Additionally, hepatic tissues from both untreated and treated mice displayed comparable normal architecture, devoid of inflammatory infiltrates, necrosis, congestion, or metastasis. Concerning the lungs, mice in the control and 0.5 mg/kg LA groups demonstrated healthy, thin alveolar septa, with no hemorrhage, fibrin deposition, or intra-alveolar and interstitial inflammatory cells. Notably, in the 1 mg/kg LA group, only 17% (one-sixth) of the mice exhibited minimal congestion in the alveolar septa, with no other structural abnormalities observed. Similarly, in the 2 mg/kg LA group, 33% (one-third) of the mice showed minimal congestion in the alveolar septa, without additional alterations. These minimal changes were not accompanied by inflammation, hemorrhage, or other structural alterations and occurred in only 1/6 and 2/6 animals in the 1 mg/kg and 2 mg/kg groups, respectively. They were considered incidental findings and do not indicate treatment-related toxicity. Finally, the examination of the mucosal lining of the nasal cavity revealed no significant changes in the epithelium or signs of inflammation across all mice. A summary of the histopathological findings as percentage of animals in each severity grade is presented in Table 1, with individual animal scores per each major organ (heart, kidneys, spleen, liver, lungs) and tissue (nasal mucosa) are provided in Supplementary Table S1 for full transparency.

These histological observations complete the subacute toxicity assessment of various Larixyl acetate doses, with the selected concentrations supporting a favorable safety profile both systemically and locally under the tested timeframe.

## 4. Discussion

Among many other candidates, Larixyl acetate was chosen for its well-established safety, specificity profile [6] and anti-inflammatory properties [4,8,26]. Furthermore, several murine in vivo studies have employed Larixyl acetate therapeutically to ameliorate pressure overload-induced heart failure [10] and to protect against systemic endothelial dysfunction following TBI [9]. Both studies, however, have administered Larixyl acetate systemically via the intraperitoneal route. Because no other routes of administration have been reported or tested in the literature, and because no toxicological studies have examined the safety profile of Larixyl acetate, we sought to evaluate an alternative route that better aligns with the upper airway treatment regimen. As the primary mode of drug delivery is local, topically administered via intranasal sprays, we aimed to specifically examine the phenotypic toxicity profile of three intranasally administered Larixyl acetate doses using an in vivo 30-day subacute toxicity model. In this model, the key elements of the OECD Test Guideline 412 for subacute inhalation toxicity were followed [17].

Consequently, three doses of Larixyl acetate (0.5, 1, and 2 mg/kg) were tested, with the highest dose being 2 mg/kg, in accordance with previously published work on Ca^2+^ channel blockers (CCBs) given via the same route [20]. Throughout the model, no statistically significant differences were observed in recorded body weight, indicating that Larixyl acetate had no detrimental effects on overall anthropometric measurements. Additionally, at the end of the model, we harvested several key organs that are responsive to drug-induced abnormalities, including hypertrophy and atrophy. The calculated organ-to-body ratios for the heart, kidneys, liver, lungs, spleen, and brain showed no statistically significant alterations in organ size or weight. These findings establish the first layer for Larixyl acetate’s toxicity assessment.

Furthermore, a prompt assessment of systemic changes reflected in hematological markers was performed by conducting a complete blood count (CBC) on blood samples collected at the time of sacrifice. This specific assessment is crucial, as topically or locally administered drugs tend to be absorbed into the body’s circulation over time, as is the case with intranasal corticosteroids (INCSs) [27]. This absorption often results in hidden effects that become evident much later in life, especially with extended use [28]. In the case of Larixyl acetate, limited fluctuations in WBC or RBC profiles were observed across all treatment groups. These results suggest the absence of drug-induced immunosuppressive, inflammatory, or thromboembolic side effects [29]. The lack of these abnormalities further supports Larixyl acetate’s subacute toxicity profile.

A comprehensive biochemical analysis reflecting key liver and kidney enzymes, including ALP, AST, ALT, TBIL, uric acid, and creatinine, was also performed. Measuring enzymatic levels in the isolated serum samples revealed minute, statistically insignificant differences across all groups. Although a few individual animals showed higher ALT and total bilirubin values, these remained well within the published normal physiological ranges for female BALB/c mice [30] and did not reach statistical significance across groups. There was also no corresponding histopathological evidence of hepatotoxicity in liver sections from any treatment group. These occasional outliers are therefore considered to reflect normal biological variation rather than rare treatment-related hepatic toxicity. These findings provided deeper insights into the inner workings of these organs, specifically the two major ones responsible for detoxifying, processing, and excreting drugs. Elevated levels often indicate a significantly compromised functional profile, indicative of internal tissue damage and cellular death [31,32]. As such, these findings suggest no observable nephrotoxic or hepatotoxic effects under the conditions tested.

As part of our comprehensive final evaluation to identify any overlooked indicators of organ or tissue damage, we harvested several organs post-mortem for their valuable systemic insights, including the heart, kidneys, spleen, lungs, and liver. To determine any localized irregularities at the drug administration site, mice skulls were collected, decalcified, fixed, and sectioned to examine the nasal mucosa. Our rigorous histological assessment revealed no clinically significant alterations in tissue morphology, with no indications of inflammation, cellular hypertrophy, or atrophy. Notably, minimal alveolar septal congestion was observed in the lungs of both the mid- and high-dose groups. Although these findings appear to be dose-dependent, they do not have significant implications for the toxicity of Larixyl acetate, as transient permeability alterations have been previously documented in pulmonary models following inhibition or deletion of TRPC6 [14]. The reversible nature of these changes was demonstrated in air–liquid interface (ALI) models, thereby excluding chronicity and supporting their insignificance [33]. Accordingly, these incidental findings do not compromise the safety profile of Larixyl acetate at doses of 1 mg/kg and 2 mg/kg, as the mild severity permits the continued development of the therapeutic approach [34]. Compared with other TRPC6 inhibitors, the three tested doses of Larixyl acetate showed a comparable or more favorable short-term toxicity profile via the intranasal route in the present evaluation. Notably, previous selective and non-selective TRPC inhibitors like SKF-96365, SAR7334, BI 749327, and SH045 were employed intranasally, orally, and intraperitoneally at doses of 16–30 mg/kg in murine models of inflammatory disease (e.g., allergic rhinitis) without any reported systemic toxicity or mucosal irritation, even when given for prolonged durations [12,16,18,35]. Similarly, Larixyl acetate itself has been used at 5 mg/kg intraperitoneally or daily in models of traumatic brain injury and pressure overload-induced heart failure with no adverse effects noted [9,10]. In contrast, the present sub-acute intranasal study of Larixyl acetate at 0.5–2 mg/kg (doses 2.5- to 10-fold lower than the established safe intraperitoneal therapeutic range) revealed no statistically significant alterations in any toxicity parameter, supporting its favorable short-term toxicity profile for potential airway applications.

The collective findings from our study investigating the phenotypic toxicity of intranasally administered Larixyl acetate indicated a favorable subacute toxicity profile, even at a higher dose (2 mg/kg). This dose range is consistent with those used for other intranasally administered Ca^2+^-channel modulators in murine airway models [20].

## 5. Limitations and Future Perspectives

The present study has several limitations that should be considered when interpreting the results. It was designed as a phenotypic toxicological evaluation and did not include mechanistic investigations of TRPC6 expression, activity, or downstream signaling. No inflammatory mediators, cytokines, or epithelial barrier markers were assessed. The study, therefore, cannot support any mechanistic claims regarding TRPC6 inhibition.

Pharmacokinetic and exposure data on local versus systemic absorption, serum or tissue levels of Larixyl acetate, and its metabolism or clearance were not obtained. The absence of pharmacokinetic data prevents any conclusions regarding target engagement or whether the administered doses achieved pharmacologically relevant concentrations at the site of action. These measurements were not performed in the present study because it was designed as a basic phenotypic subacute toxicity screen focused on standard OECD-guided endpoints (clinical observations, body and organ weights, hematology, clinical chemistry, and histopathology). Inclusion of comprehensive pharmacokinetic analysis would have required additional satellite groups, larger animal numbers, and specialized bioanalytical methods, which were beyond the scope and resources of this foundational toxicity assessment. Similar approaches are commonly adopted in early-stage phenotypic toxicity studies, where the primary objective is to identify overt toxicity signals rather than fully characterize absorption, distribution, metabolism, and excretion profiles.

Additionally, the sample size of (*n* = 6) per group provides limited statistical power to detect subtle changes in complete blood count parameters, serum biochemical markers, and organ-to-body weight ratios. Furthermore, the absence of statistical significance (*p* > 0.05) does not constitute evidence of the absence of an effect or proof of safety. Evidently, the one-way ANOVA revealed a statistically significant overall treatment effect (*p* < 0.05) with a substantial effect size ($R^{2}=0.34$) in organ-to-body weight ratios of the kidneys and brain, as well as in ALP levels, thus indicating that the treatment accounted for 33–34% of the total variance. However, pairwise comparisons via Bonferroni post hoc analysis failed to identify statistically significant differences between specific groups (*p* > 0.05). This scenario typically arises from the conservative nature of the Bonferroni adjustment in the presence of high intra-group biological variability and a modest sample size, which limited the power to isolate localized differences despite a strong global treatment effect.

The observation period was limited to 30 days in accordance with OECD Test Guideline 412 for subacute toxicity assessment. The study also lacked a dedicated recovery phase, which is standard in many OECD-aligned toxicity studies. Consequently, the reversibility of any potential effects and the possibility of delayed toxicity could not be evaluated. Future studies incorporating longer observation periods and dedicated recovery phases will be essential to fully characterize the long-term safety profile of intranasally administered Larixyl acetate.

OECD Test Guideline 412 specifically addresses inhalation toxicity via aerosol generation and exposure in chambers with defined aerosol characterization, exposure conditions, and respiratory deposition modeling. In contrast, the present study used intranasal instillation (pipetting of 20 µL under anesthesia) as a practical surrogate for local upper-airway delivery rather than true inhalation toxicity testing.

Notably, TRPC6 inhibition is known to influence cardiovascular function, and intranasal delivery of other cardiovascular agents has been shown to enable rapid systemic effects through the rich nasal vascularization and direct drainage to the right heart, dedicated functional cardiovascular assessments such as echocardiography or blood pressure monitoring, were not performed in this basic phenotypic toxicity study [19,27].

## 6. Conclusions

Ultimately, this study provides phenotypic insights into the subacute toxicological profile of intranasally administered Larixyl acetate in female BALB/c mice. In this study, multiple parameters were assessed for any compound-related detrimental effects, both systemically and locally, following the general principles of the OECD Test Guideline 412. The results showed no statistically significant toxicity under the tested conditions, with minimal changes observed in any of the evaluated parameters. These data provide a foundation for future efficacy and chronic toxicity studies of Larixyl acetate in TRPC6-related respiratory conditions.

## Figures and Tables

**Table 1:** Summary of the incidence and severity of histopathological changes in organs and tissues following 30-day intranasal administration of Larixyl acetate. Data are presented as the percentage of animals per group showing each finding (*n* = 6 mice/group).

Organs and Tissues	LA (mg/kg)			
	Control	0.5	1	2
Heart	Within normal limits (100%)	Within normal limits (100%)	Within normal limits (100%)	Within normal limits (100%)
Kidneys	Within normal limits (100%)	Within normal limits (100%)	Within normal limits (100%)	Within normal limits (100%)
Spleen	Within normal limits (100%)	Within normal limits (100%)	Within normal limits (100%)	Within normal limits (100%)
Liver	Within normal limits (100%)	Within normal limits (100%)	Within normal limits (100%)	Within normal limits (100%)
Lungs	Within normal limits (100%)	Within normal limits (100%)	Within normal limits (83%), Minimal (17%)	Within normal limits (67%), Minimal (33%)
Nasal mucosa	Within normal limits (100%)	Within normal limits (100%)	Within normal limits (100%)	Within normal limits (100%)

## Data Availability

The original contributions presented in this study are included in the article and Supplementary Material. Further inquiries can be directed to the corresponding author.

## Supplementary Materials

The following supporting information can be downloaded at: https://www.mdpi.com/article/10.3390/jox16030100/s1, Table S1: Individual semi-quantitative histopathology scores (0–4 scale) for each mouse per major organ and tissue following 30 days of intranasal administration of Larixyl acetate; Figure S1: Raw high-resolution images of hematoxylin and eosin (H&E) stained sections (heart, kidneys, spleen, liver, lungs, and nasal mucosa).

## Abbreviations

The following abbreviations are used in this manuscript:

ALI	Air–liquid interface
ALP	Alkaline phosphatase
ALT	Alanine aminotransferase
ANOVA	Analysis of variance
AST	Aspartate aminotransferase
Ca^2+^	Calcium
CBC	Complete blood count
CRS	Chronic rhinosinusitis
CRSwNP	Chronic rhinosinusitis with nasal polyps
H&E	Hematoxylin and eosin
LA	Larixyl acetate
LTB_4_	Leukotriene B4
MCV	Mean corpuscular volume
MPV	Mean platelet volume
MCHC	Mean corpuscular hemoglobin concentration
MRSA	Methicillin-resistant *Staphylococcus aureus*
NSAIDs	Non-steroidal anti-inflammatory drugs
OECD	Organization for Economic Co-operation and Development
OVA	Ovalbumin
PBS	Phosphate-buffered saline
RBC	Red blood cell
TBI	Traumatic brain injury
TBIL	Total bilirubin
TRPC6	Transient receptor potential canonical channel 6
WBC	White blood cell

## Appendix A

### Appendix A.1

**Table A1:** Effect of LA on organ index in female mice (*n* = 6). Data are presented as mean value ± SEM, complemented by group-specific 95% confidence intervals (CIs), global one-way ANOVA *p*-values, and overall effect sizes ($R^{2}$).

Organ	Treatment Groups	Mean ± SEM	95% Confidence Interval (CI) of Difference	*p*-Value	Effect Size ($R^{2}$)
Heart	Control	0.82 ± 0.03	(Reference)	0.74	0.06
	0.5 mg/kg LA	0.84 ± 0.01	−0.13 to 0.07		
	1 mg/kg LA	0.84 ± 0.02	−0.13 to 0.07		
	2 mg/kg LA	0.82 ± 0.03	−0.11 to 0.09		
Liver	Control	4.57 ± 0.11	(Reference)	0.06	0.30
	0.5 mg/kg LA	4.38 ± 0.12	−0.33 to 0.72		
	1 mg/kg LA	4.78 ± 0.17	−0.74 to 0.31		
	2 mg/kg LA	4.30 ± 0.10	−0.26 to 0.79		
Spleen	Control	0.39 ± 0.01	(Reference)	0.14	0.24
	0.5 mg/kg LA	0.37 ± 0.02	−0.03 to 0.07		
	1 mg/kg LA	0.38 ± 0.01	−0.04 to 0.06		
	2 mg/kg LA	0.35 ± 0.01	−0.01 to 0.09		
Lungs	Control	1.41 ± 0.06	(Reference)	0.08	0.28
	0.5 mg/kg LA	1.34 ± 0.04	−0.12 to 0.27		
	1 mg/kg LA	1.23 ± 0.03	−0.02 to 0.38		
	2 mg/kg LA	1.29 ± 0.05	−0.08 to 0.32		
Kidneys	Control	1.46 ± 0.04	(Reference)	0.04	0.33
	0.5 mg/kg LA	1.44 ± 0.04	−0.12 to 0.16		
	1 mg/kg LA	1.58 ± 0.03	−0.26 to 0.02		
	2 mg/kg LA	1.49 ± 0.03	−0.17 to 0.11		
Brain	Control	2.68 ± 0.03	(Reference)	0.04	0.34
	0.5 mg/kg LA	2.67 ± 0.03	−0.14 to 0.15		
	1 mg/kg LA	2.60 ± 0.05	−0.07 to 0.22		
	2 mg/kg LA	2.54 ± 0.03	−0.01 to 0.28		

### Appendix A.2

**Table A2:** Hematological parameters in female mice after intranasal administration of LA (*n* = 6). Data are presented as mean value ± SEM, complemented by group-specific 95% confidence intervals (CIs), global one-way ANOVA *p*-values, and overall effect sizes ($R^{2}$).

Parameter	Treatment Groups	Mean ± SEM	95% Confidence Interval (CI) of Difference	*p*-Value	Effect Size ($R^{2}$)
WBC ^1^ (∗10^3^/µL)	Control	0.97 ± 0.31	(Reference)	0.92	0.02
	0.5 mg/kg LA	0.80 ± 0.20	−0.70 to 1.04		
	1 mg/kg LA	0.78 ± 0.16	−0.68 to 1.07		
	2 mg/kg LA	0.85 ± 0.12	−0.75 to 0.99		
Neu ^2^ (%)	Control	6.01 ± 0.87	(Reference)	0.41	0.13
	0.5 mg/kg LA	6.70 ± 1.57	−6.15 to 4.76		
	1 mg/kg LA	6.62 ± 1.47	−6.06 to 4.84		
	2 mg/kg LA	9.00 ± 1.26	−8.45 to 2.46		
Lym ^3^ (%)	Control	89.05 ± 1.78	(Reference)	0.46	0.12
	0.5 mg/kg LA	85.72 ± 4.36	−7.88 to 14.53		
	1 mg/kg LA	91.49 ± 1.88	−13.66 to 8.76		
	2 mg/kg LA	86.85 ± 1.91	−9.01 to 13.41		
Mon ^4^ (%)	Control	1.34 ± 0.71	(Reference)	0.29	0.17
	0.5 mg/kg LA	3.51 ± 2.12	−7.00 to 2.65		
	1 mg/kg LA	0.30 ± 0.17	−3.79 to 5.86		
	2 mg/kg LA	2.07 ± 0.64	−5.56 to 4.10		
Eos ^5^ (%)	Control	3.41 ± 1.02	(Reference)	0.67	0.07
	0.5 mg/kg LA	3.96 ± 2.89	−7.05 to 5.94		
	1 mg/kg LA	1.57 ± 0.53	−4.65 to 8.33		
	2 mg/kg LA	1.95 ± 0.44	−5.04 to 7.94		
Bas ^6^ (%)	Control	0.21 ± 0.15	(Reference)	0.59	0.09
	0.5 mg/kg LA	0.10 ± 0.09	−0.28 to 0.49		
	1 mg/kg LA	0.02 ± 0.02	−0.20 to 0.57		
	2 mg/kg LA	0.14 ± 0.07	−0.31 to 0.45		
Neu ^7^ (∗10^3^/µL)	Control	0.05 ± 0.01	(Reference)	0.40	0.13
	0.5 mg/kg LA	0.04 ± 0.01	−0.04 to 0.06		
	1 mg/kg LA	0.05 ± 0.02	−0.05 to 0.05		
	2 mg/kg LA	0.07 ± 0.01	−0.08 to 0.03		
Lym ^8^ (∗10^3^/µL)	Control	0.88 ± 0.29	(Reference)	0.91	0.03
	0.5 mg/kg LA	0.71 ± 0.18	−0.62 to 0.95		
	1 mg/kg LA	0.71 ± 0.14	−0.62 to 0.95		
	2 mg/kg LA	0.74 ± 0.10	−0.66 to 0.92		
Mon ^9^ (∗10^3^/µL)	Control	0.02 ± 0.02	(Reference)	0.47	0.12
	0.5 mg/kg LA	0.04 ± 0.03	−0.09 to 0.05		
	1 mg/kg LA	0.002 ± 0.002	−0.05 to 0.09		
	2 mg/kg LA	0.02 ± 0.01	−0.07 to 0.07		
Eos ^10^ (∗10^3^/µL)	Control	0.03 ± 0.01	(Reference)	0.40	0.13
	0.5 mg/kg LA	0.01 ± 0.01	−0.12 to 0.39		
	1 mg/kg LA	0.01 ± 0.01	−0.12 to 0.39		
	2 mg/kg LA	0.02 ± 0.01	−0.17 to 0.34		
Bas ^11^ (∗10^3^/µL)	Control	0 ± 0	(Reference)	0.58	0.09
	0.5 mg/kg LA	0.002 ± 0.002	−0.01 to 0.003		
	1 mg/kg LA	0 ± 0	−0.005 to 0.005		
	2 mg/kg LA	0.002 ± 0.002	−0.01 to 0.003		
RBC ^12^ (∗10^6^/µL)	Control	7.63 ± 0.31	(Reference)	0.48	0.11
	0.5 mg/kg LA	7.90 ± 0.14	−1.21 to 0.67		
	1 mg/kg LA	7.58 ± 0.25	−0.90 to 0.99		
	2 mg/kg LA	8.02 ± 0.17	−1.33 to 0.55		
HGB ^13^ (g/dL)	Control	13.54 ± 0.47	(Reference)	0.24	0.19
	0.5 mg/kg LA	13.93 ± 0.18	−1.79 to 1.00		
	1 mg/kg LA	13.14 ± 0.38	−1.00 to 1.78		
	2 mg/kg LA	14.07 ± 0.24	−1.93 to 0.86		
HCT ^14^ (%)	Control	39.38 ± 1.61	(Reference)	0.62	0.08
	0.5 mg/kg LA	40.13 ± 0.69	−5.75 to 4.25		
	1 mg/kg LA	38.58 ± 1.39	−4.20 to 5.80		
	2 mg/kg LA	40.75 ± 0.92	−6.36 to 3.63		
MCV ^15^ (fL)	Control	51.55 ± 0.28	(Reference)	0.05	0.32
	0.5 mg/kg LA	50.67 ± 0.09	−0.10 to 1.63		
	1 mg/kg LA	50.85 ± 0.23	−0.17 to 1.57		
	2 mg/kg LA	50.80 ± 0.09	−0.12 to 1.62		
MCHC ^16^ (g/dL)	Control	34.42 ± 0.29	(Reference)	0.16	0.22
	0.5 mg/kg LA	35.20 ± 0.48	−2.16 to 0.59		
	1 mg/kg LA	34.10 ± 0.28	−1.06 to 1.69		
	2 mg/kg LA	34.57 ± 0.23	−1.53 to 1.23		
PLT ^17^ (∗10^3^/µL)	Control	411.28 ± 28.87	(Reference)	0.31	0.16
	0.5 mg/kg LA	462.20 ± 13.18	−164.0 to 62.21		
	1 mg/kg LA	481.15 ± 32.34	−183.0 to 43.26		
	2 mg/kg LA	469.75 ± 30.56	−171.6 to 54.66		
MPV ^18^ (fL)	Control	6.66 ± 0.26	(Reference)	0.30	0.16
	0.5 mg/kg LA	6.33 ± 0.12	−0.49 to 1.15		
	1 mg/kg LA	6.17 ± 0.16	−0.33 to 1.31		
	2 mg/kg LA	6.58 ± 0.22	−0.74 to 0.90		

^1^: White blood cell, ^2^: Percentage of neutrophils, ^3^: Percentage of lymphocytes, ^4^: Percentage of monocytes, ^5^: Percentage of eosinophils, ^6^: Percentage of basophils, ^7^: Neutrophil count, ^8^: Lymphocyte count, ^9^: Monocyte count, ^10^: Eosinophil count, ^11^: Basophil count, ^12^: Red blood cell, ^13^: Hemoglobin, ^14^: Hematocrit, ^15^: Mean corpuscular volume, ^16^: Mean corpuscular hemoglobin, ^17^: Platelet count, ^18^: Mean platelet volume.

### Appendix A.3

**Table A3:** Biochemical parameters in female mice after intranasal administration of LA (*n* = 6). Data are presented as mean value ± SEM, complemented by group-specific 95% confidence intervals (CIs), global one-way ANOVA *p*-values, and overall effect sizes ($R^{2}$).

Parameter	Treatment Groups	Mean ± SEM	95% Confidence Interval (CI) of Difference	*p*-Value	Effect Size ($R^{2}$)
AST ^1^ (U/L)	Control	218.58 ± 41.35	(Reference)	0.13	0.24
	0.5 mg/kg LA	254.33 ± 51.86	−218.9 to 147.4		
	1 mg/kg LA	212.21 ± 35.39	−176.7 to 189.5		
	2 mg/kg LA	351.10 ± 46.62	−315.6 to 50.58		
ALT ^2^ (U/L)	Control	52.94 ± 3.43	(Reference)	0.25	0.18
	0.5 mg/kg LA	50.49 ± 10.46	−26.34 to 31.24		
	1 mg/kg LA	45.44 ± 6.678	−21.29 to 36.29		
	2 mg/kg LA	65.47 ± 5.28	−41.32 to 16.26		
ALP ^3^ (U/L)	Control	459.23 ± 17.62	(Reference)	0.04	0.34
	0.5 mg/kg LA	392.49 ± 17.51	−24.65 to 158.1		
	1 mg/kg LA	479.44 ± 29.22	−111.6 to 71.18		
	2 mg/kg LA	476.61 ± 21.88	−108.8 to 74.01		
T-Bil ^4^ (mg/dL)	Control	0.10 ± 0.01	(Reference)	0.35	0.15
	0.5 mg/kg LA	0.07 ± 0.004	−0.05 to 0.11		
	1 mg/kg LA	0.12 ± 0.04	−0.10 to 0.07		
	2 mg/kg LA	0.08 ± 0	−0.06 to 0.10		
CRE ^5^ (mg/dL)	Control	0.27 ± 0.01	(Reference)	0.21	0.20
	0.5 mg/kg LA	0.28 ± 0.02	−0.05 to 0.04		
	1 mg/kg LA	0.28 ± 0.01	−0.054 to 0.04		
	2 mg/kg LA	0.31 ± 0.01	−0.08 to 0.01		
UA ^6^ (mg/dL)	Control	3.28 ± 0.31	(Reference)	0.11	0.25
	0.5 mg/kg LA	3.38 ± 0.24	−1.13 to 0.93		
	1 mg/kg LA	3.27 ± 0.12	−1.01 to 1.04		
	2 mg/kg LA	4.05 ± 0.28	−1.79 to 0.26		

^1^: Aspartate aminotransferase, ^2^: Alanine aminotransferase, ^3^: Alkaline phosphatase, ^4^: Total bilirubin, ^5^: Creatinine, ^6^: Uric acid.
