# Supplementary material for: Phenotypic Subacute Toxicity Assessment of Intranasally Administered Larixyl Acetate: Implications for Potential Airway Applications
Source: J Xenobiot. 2026 Jun 1;16(3):100. doi: 10.3390/jox16030100 (PMC13301338; doi:10.3390/jox16030100)
Supplement: Supplementary file 1 [file jox-16-00100-s001.zip › Supplementary Table S1.pdf]

**Supplementary Table S1:** Individual semi-quantitative histopathology scores (0–4 scale) for each mouse per major organ or tissue following 30-days of intranasal administration of Larixyl acetate.

| Mouse ID | Group (mg/kg) | Heart | Kidneys | Spleen | Liver | Lungs | Nasal mucosa |
|---|---|---|---|---|---|---|---|
| M1 C | Control (PBS treated) | 0 | 0 | 0 | 0 | 0 | 0 |
| M2 C | | 0 | 0 | 0 | 0 | 0 | 0 |
| M3 C | | 0 | 0 | 0 | 0 | 0 | 0 |
| M4 C | | 0 | 0 | 0 | 0 | 0 | 0 |
| M5 C | | 0 | 0 | 0 | 0 | 0 | 0 |
| M6 C | | 0 | 0 | 0 | 0 | 0 | 0 |
| M1 0.5 LA | Larixyl acetate-treated (0.5 mg/kg) | 0 | 0 | 0 | 0 | 0 | 0 |
| M2 0.5 LA | | 0 | 0 | 0 | 0 | 0 | 0 |
| M3 0.5 LA | | 0 | 0 | 0 | 0 | 0 | 0 |
| M4 0.5 LA | | 0 | 0 | 0 | 0 | 0 | 0 |
| M5 0.5 LA | | 0 | 0 | 0 | 0 | 0 | 0 |
| M6 0.5 LA | | 0 | 0 | 0 | 0 | 0 | 0 |
| M1 1 LA | Larixyl acetate-treated (1 mg/kg) | 0 | 0 | 0 | 0 | 0 | 0 |
| M2 1 LA | | 0 | 0 | 0 | 0 | **1** | 0 |
| M3 1 LA | | 0 | 0 | 0 | 0 | 0 | 0 |
| M4 1 LA | | 0 | 0 | 0 | 0 | 0 | 0 |
| M5 1 LA | | 0 | 0 | 0 | 0 | 0 | 0 |
| M6 1 LA | | 0 | 0 | 0 | 0 | 0 | 0 |
| M1 2 LA | Larixyl acetate-treated (2 mg/kg) | 0 | 0 | 0 | 0 | 0 | 0 |
| M2 2 LA | | 0 | 0 | 0 | 0 | **1** | 0 |
| M3 2 LA | | 0 | 0 | 0 | 0 | 0 | 0 |
| M4 2 LA | | 0 | 0 | 0 | 0 | 0 | 0 |
| M5 2 LA | | 0 | 0 | 0 | 0 | **1** | 0 |
| M6 2 LA | | 0 | 0 | 0 | 0 | 0 | 0 |

* Scoring system: 0 = Within normal limits, 1 = Minimal, 2 = Slight, 3 = Moderate, 4 = Severe.
