# Supplementary figures and images for: Phenotypic Subacute Toxicity Assessment of Intranasally Administered Larixyl Acetate: Implications for Potential Airway Applications
Source: J Xenobiot. 2026 Jun 1;16(3):100. doi: 10.3390/jox16030100 (PMC13301338; doi:10.3390/jox16030100)

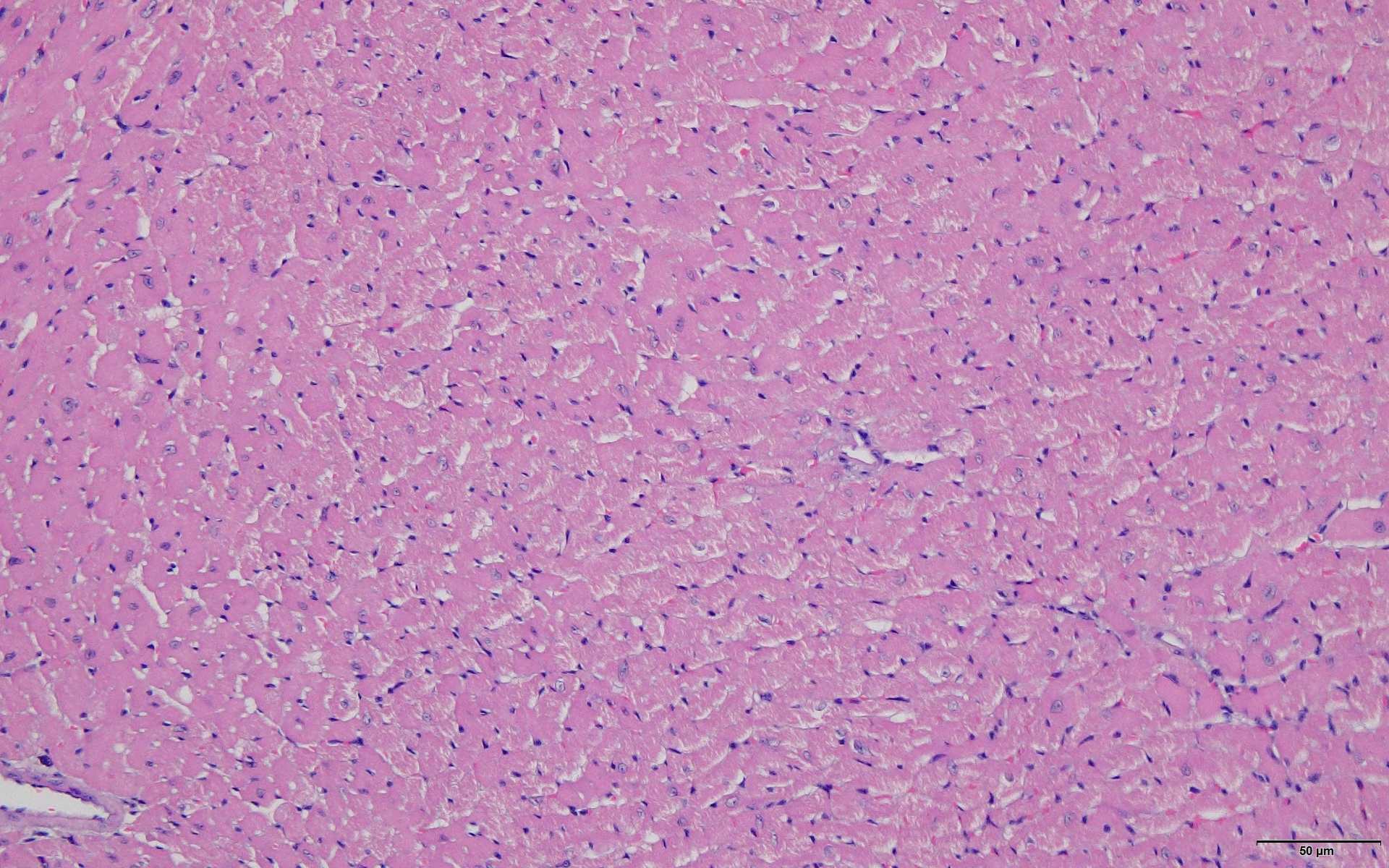

Supplement: Supplementary file 1 [file jox-16-00100-s001.zip › Figure S1/Heart h&E images/0.5 mg heart 20x.png]

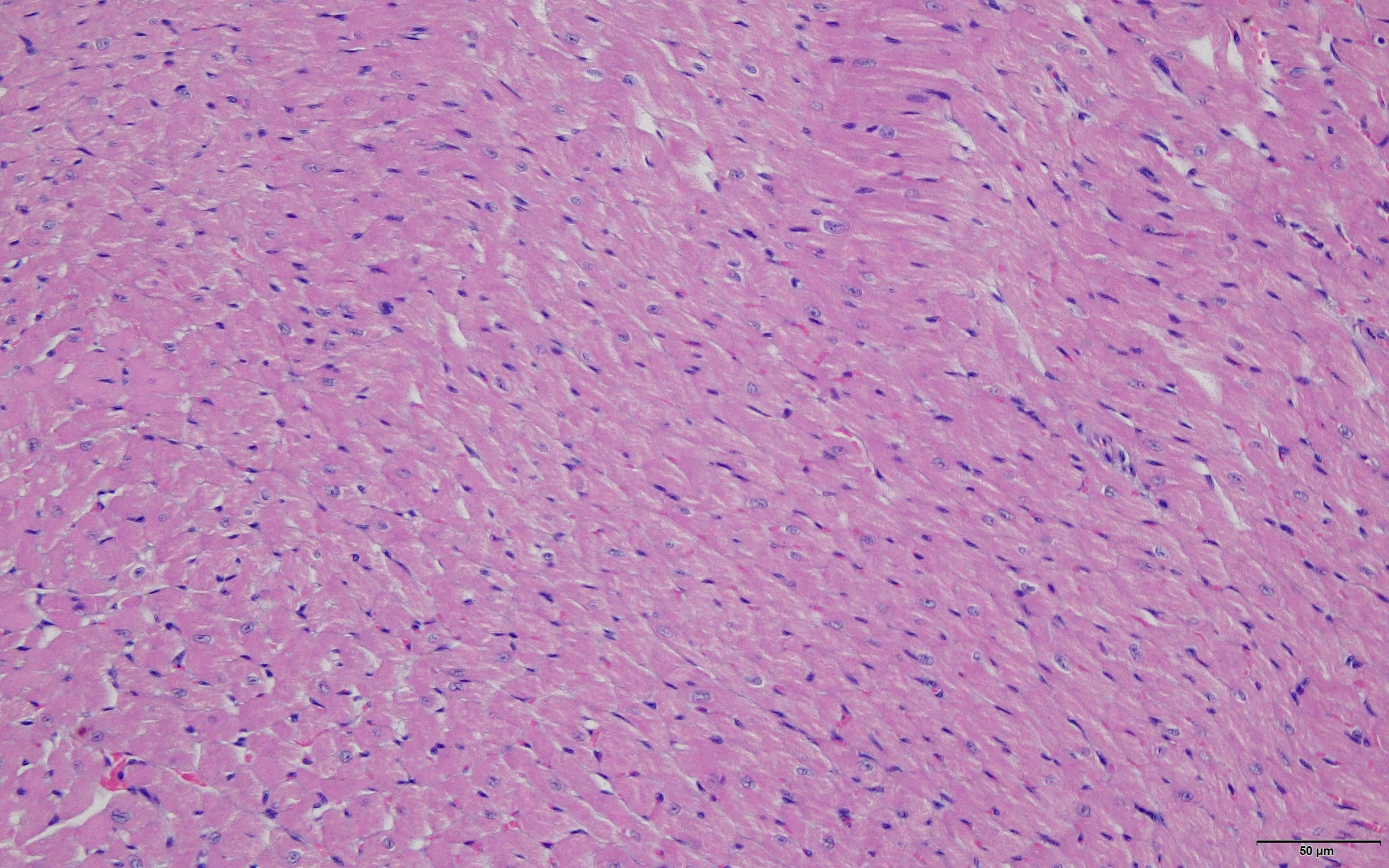

Supplement: Supplementary file 1 [file jox-16-00100-s001.zip › Figure S1/Heart h&E images/1 mg heart 20x.png]

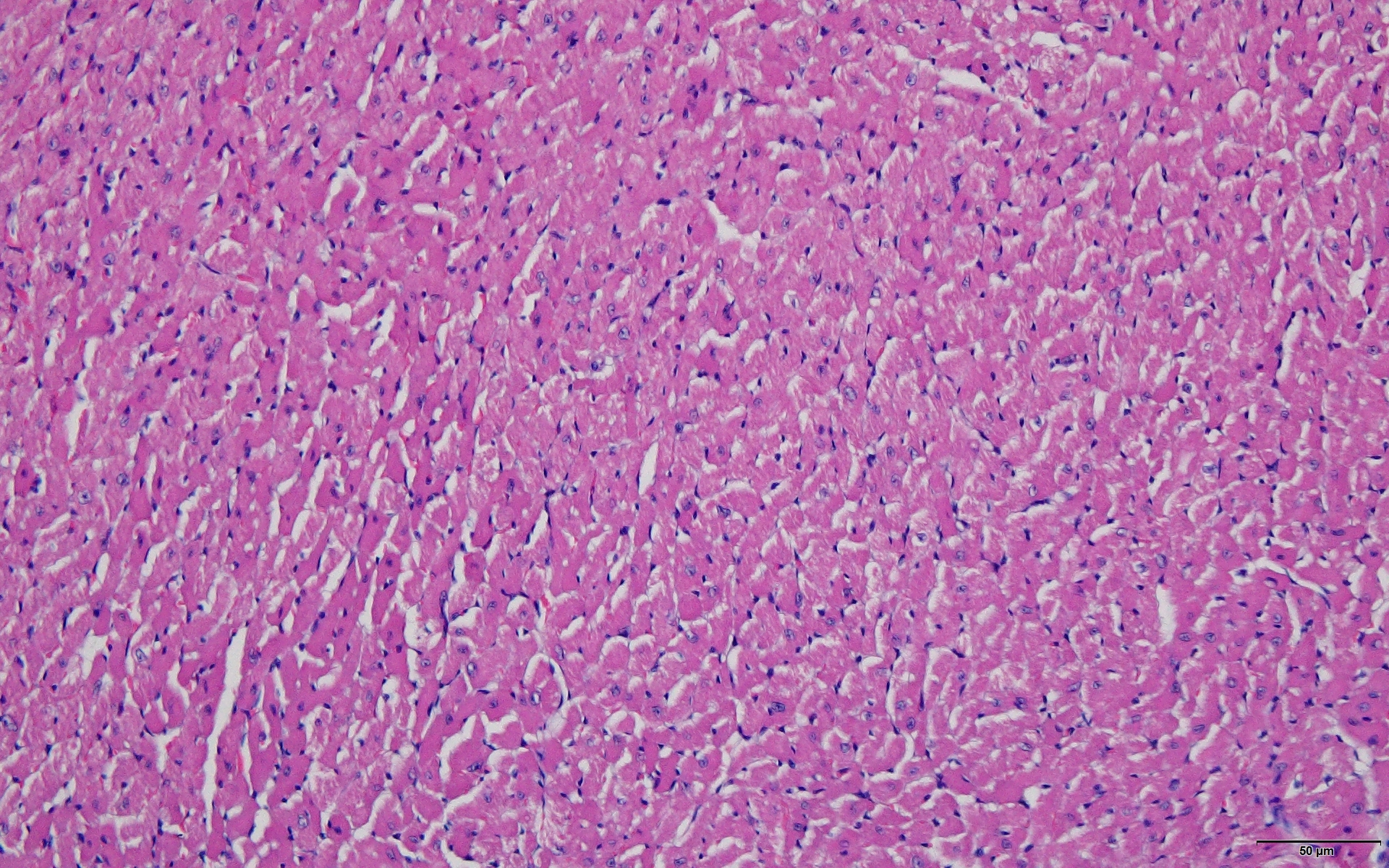

Supplement: Supplementary file 1 [file jox-16-00100-s001.zip › Figure S1/Heart h&E images/2 mg heart 20x.png]

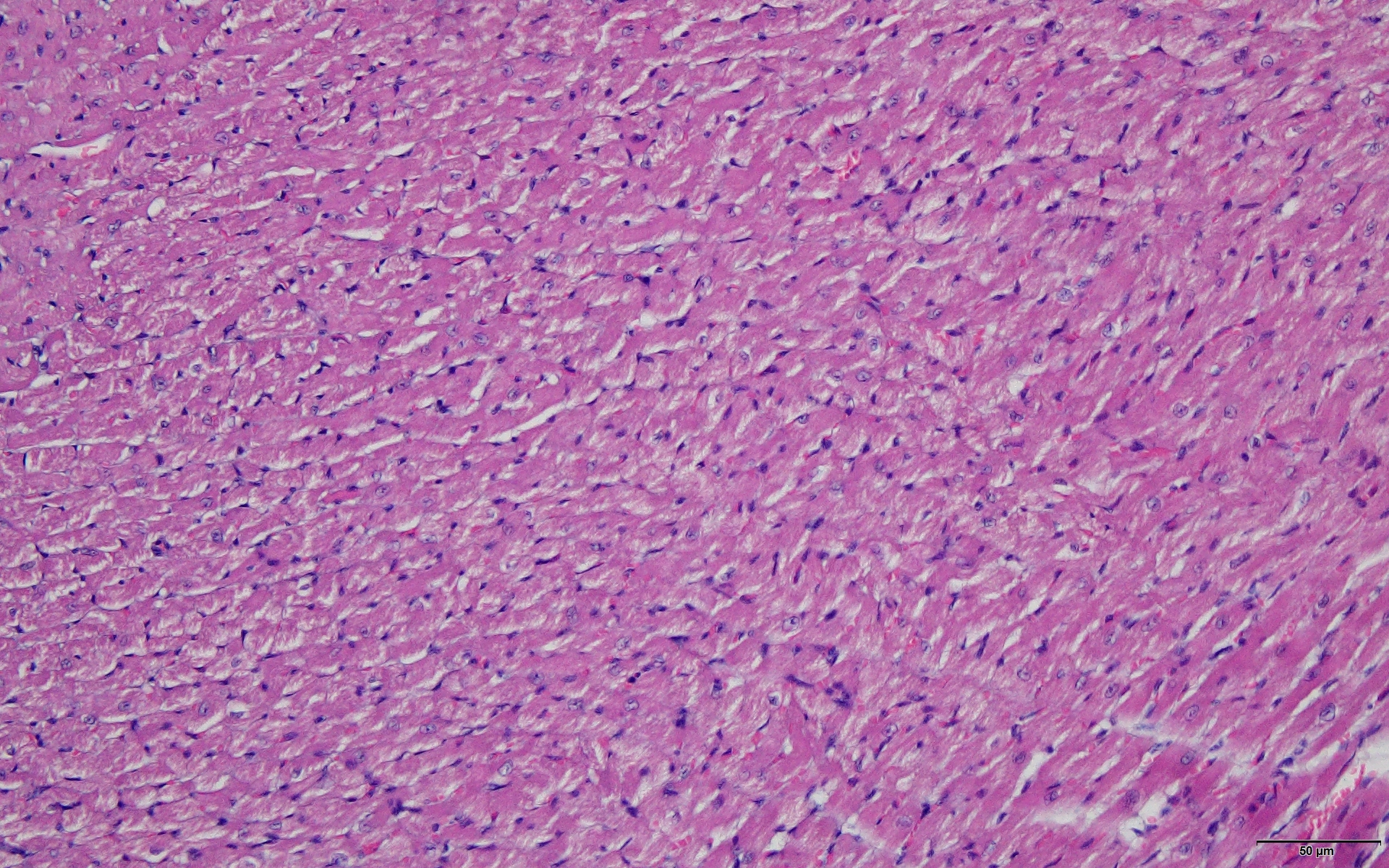

Supplement: Supplementary file 1 [file jox-16-00100-s001.zip › Figure S1/Heart h&E images/control heart 20x.png]

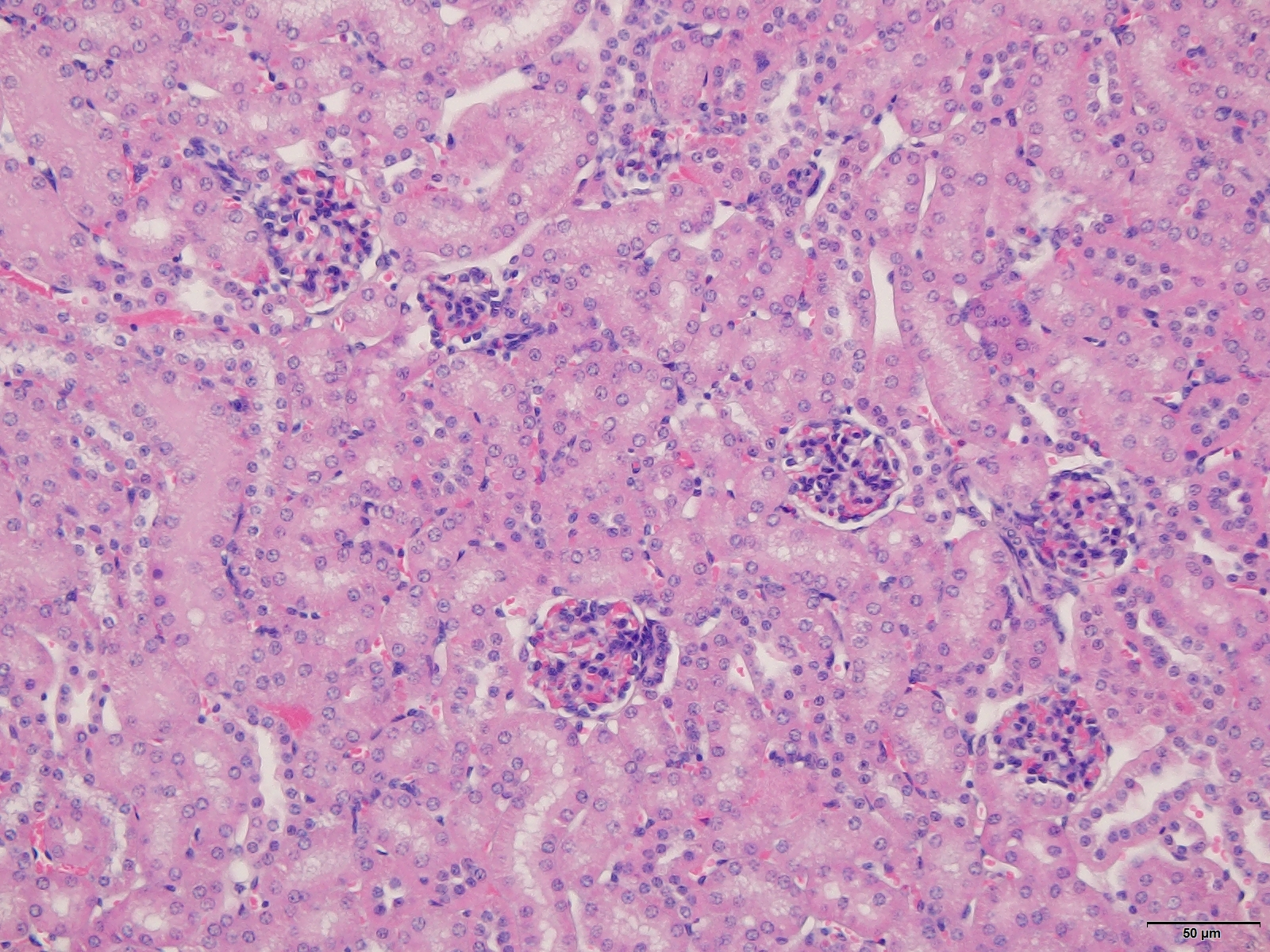

Supplement: Supplementary file 1 [file jox-16-00100-s001.zip › Figure S1/Kidneys h&E images/0.5 mg kidney 20x.png]

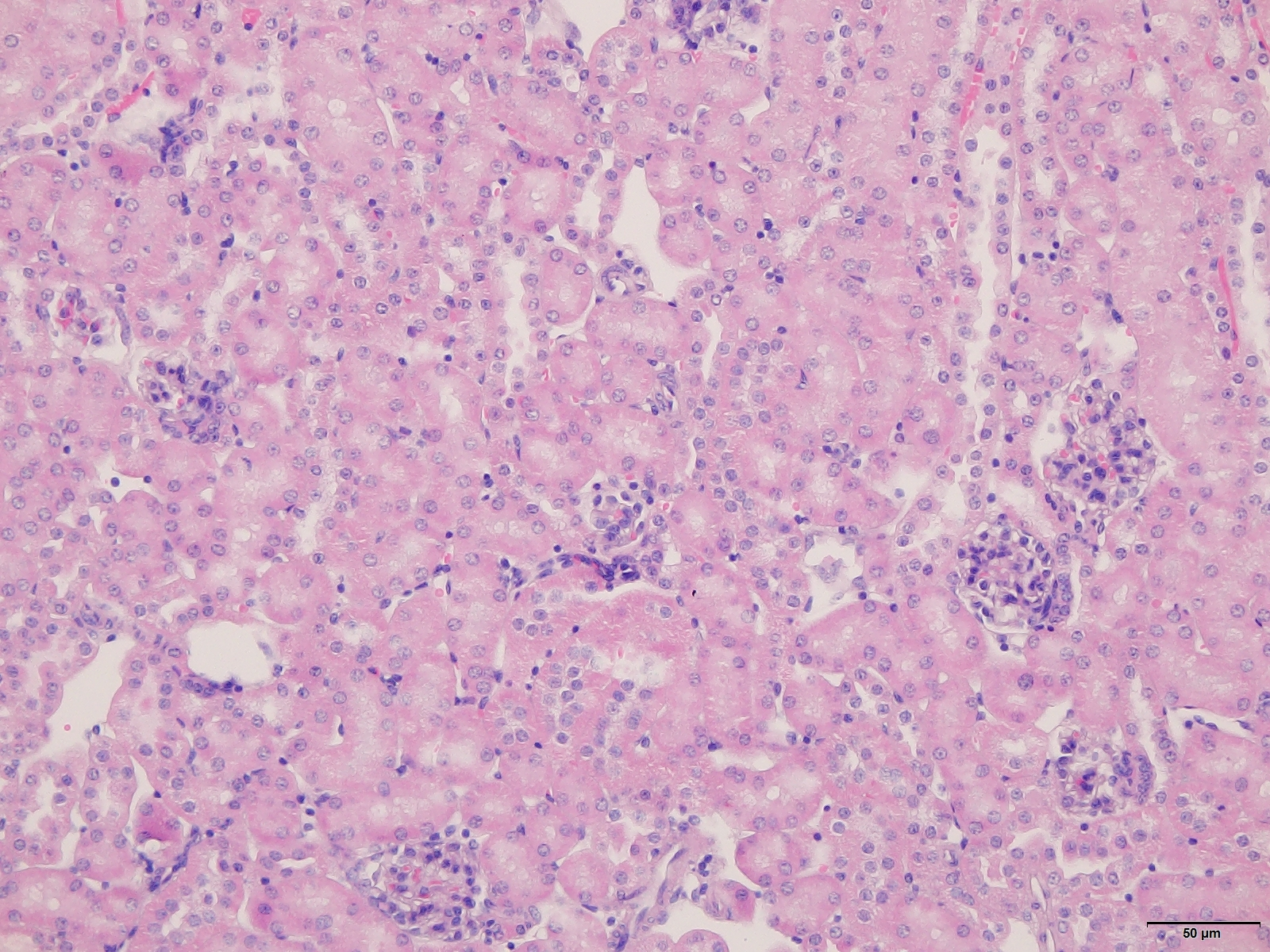

Supplement: Supplementary file 1 [file jox-16-00100-s001.zip › Figure S1/Kidneys h&E images/1 mg kidney 20x.png]

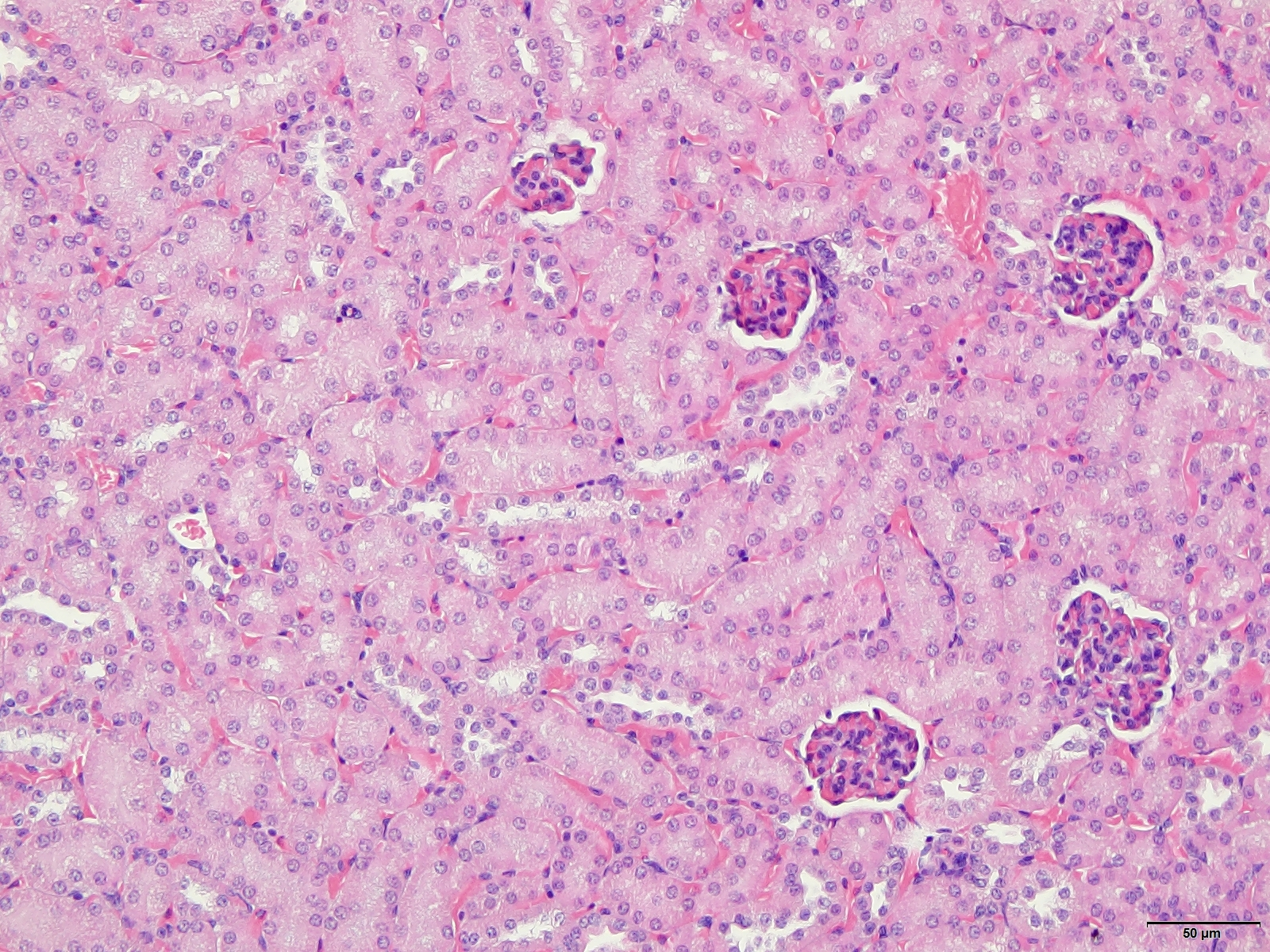

Supplement: Supplementary file 1 [file jox-16-00100-s001.zip › Figure S1/Kidneys h&E images/2 mg kidney 20x.png]

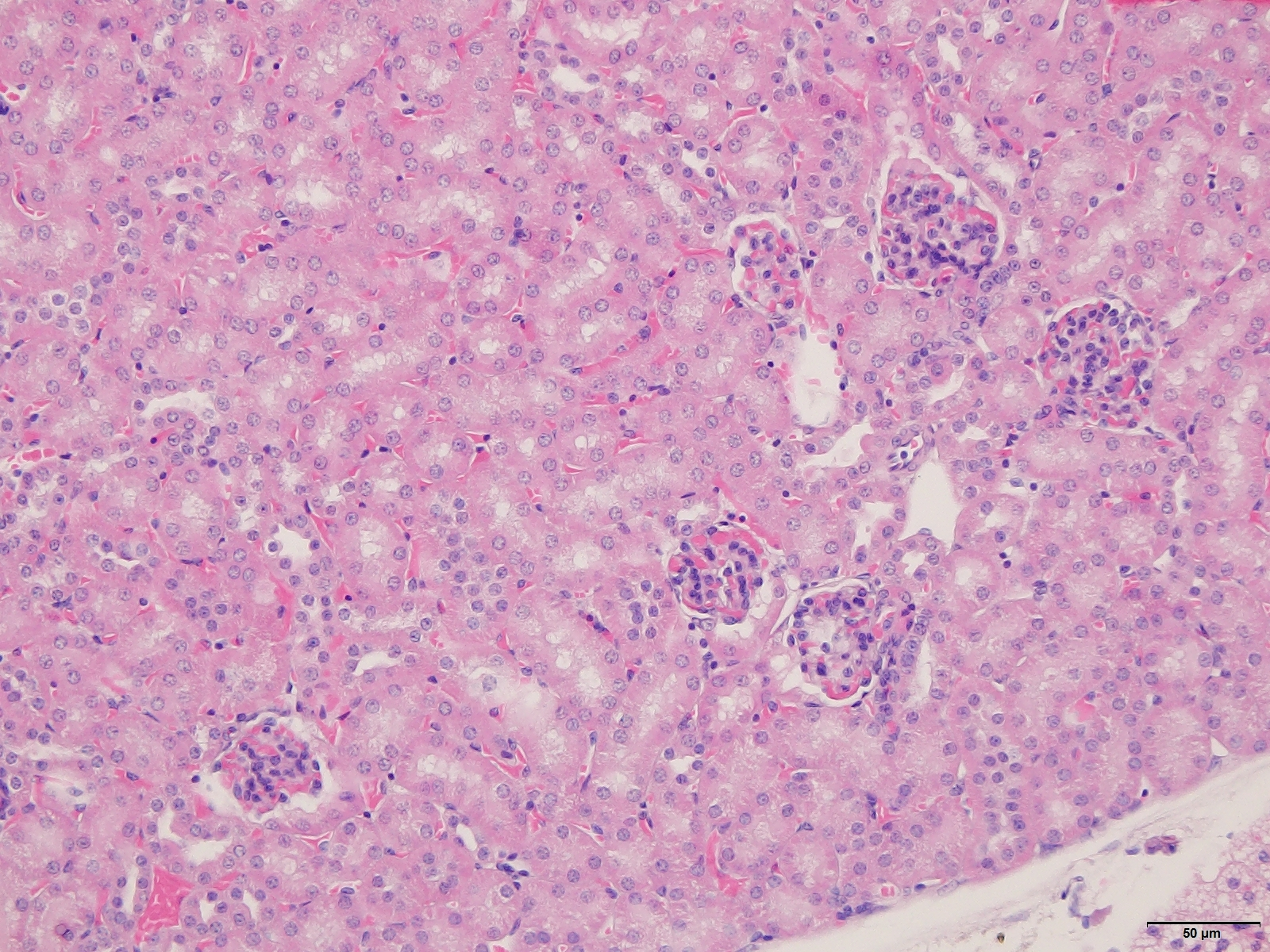

Supplement: Supplementary file 1 [file jox-16-00100-s001.zip › Figure S1/Kidneys h&E images/control kidney 20x.png]

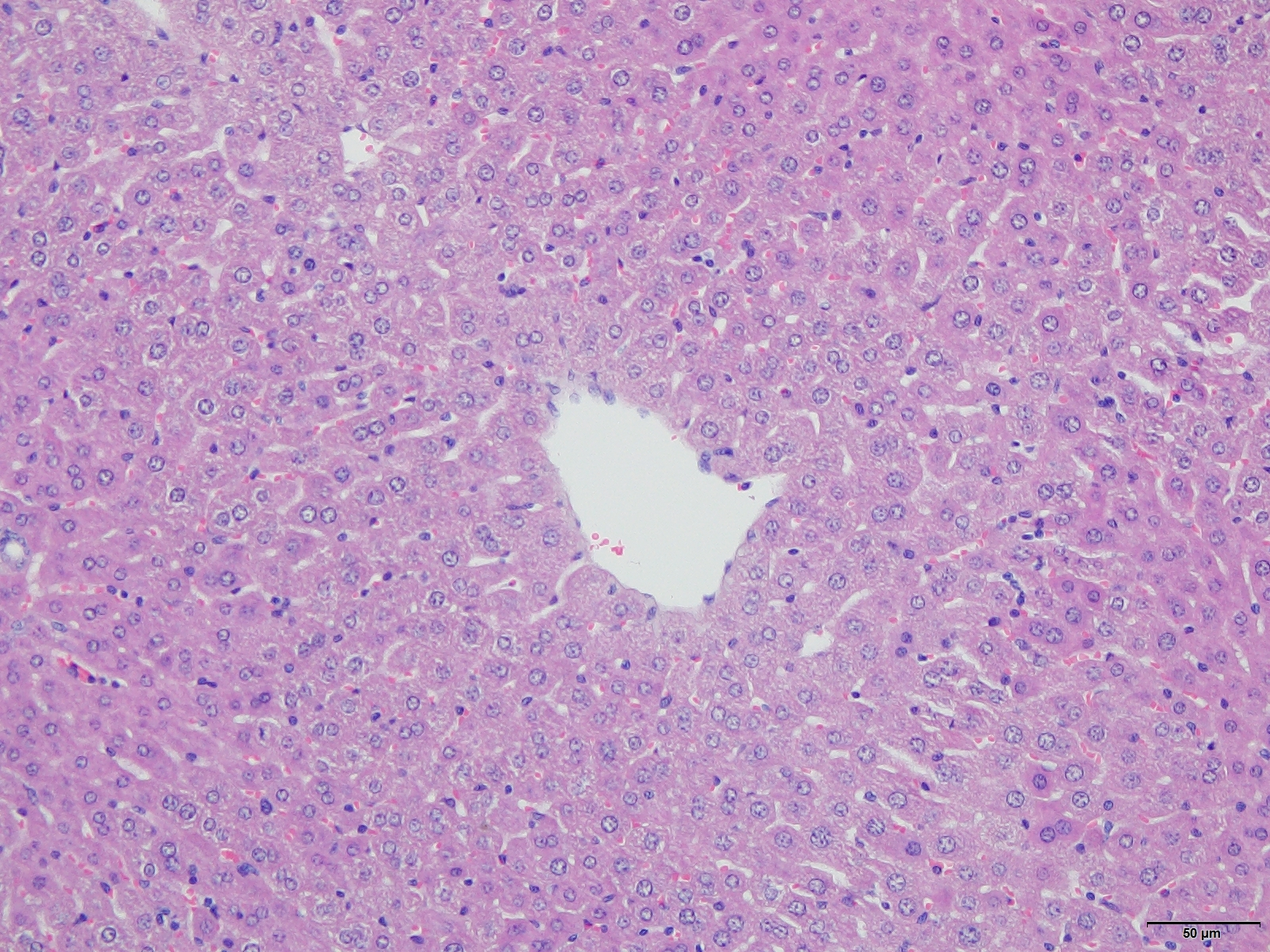

Supplement: Supplementary file 1 [file jox-16-00100-s001.zip › Figure S1/Liver h&E images/0.5 mg liver 20x.png]

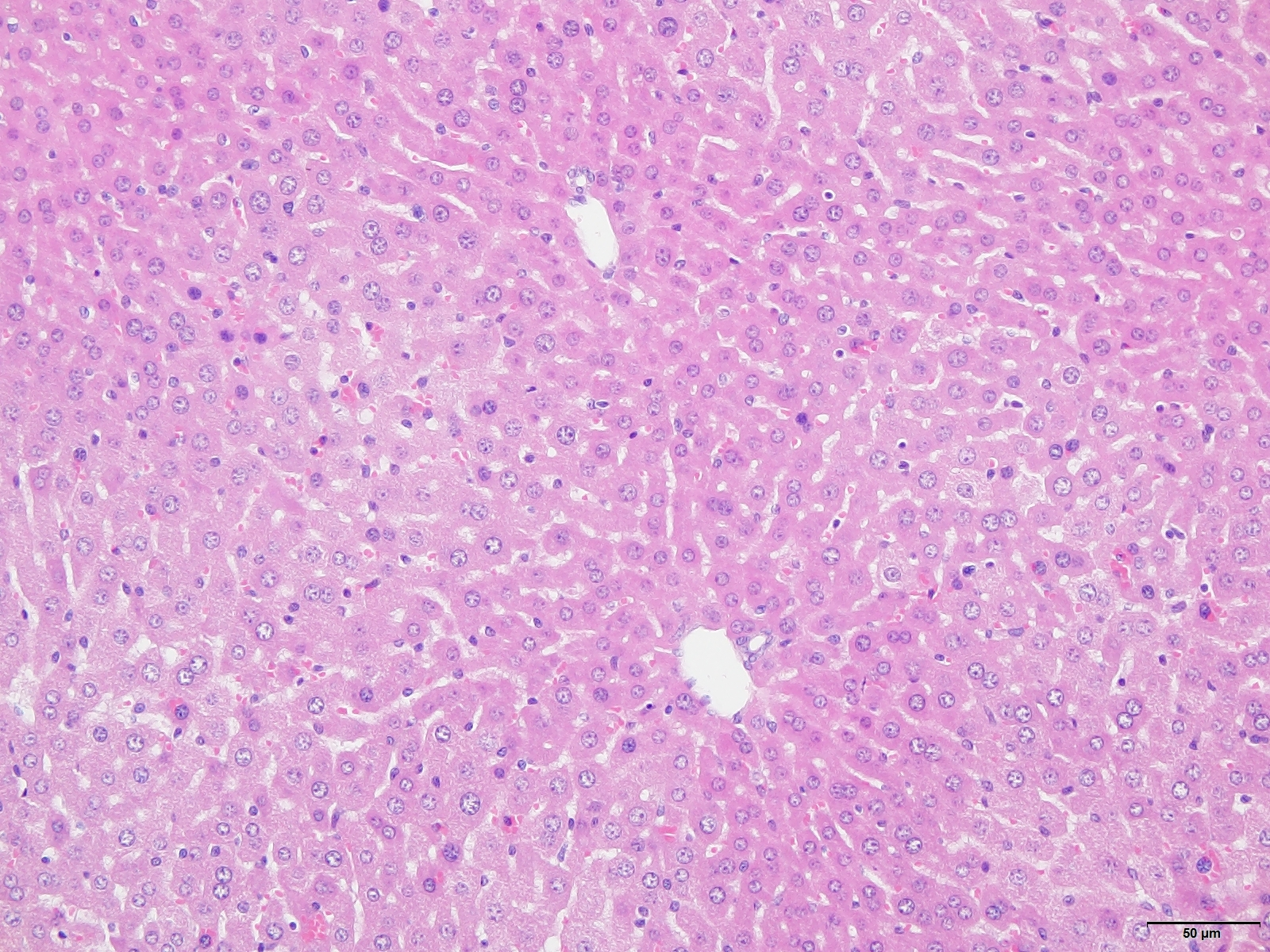

Supplement: Supplementary file 1 [file jox-16-00100-s001.zip › Figure S1/Liver h&E images/1 mg liver 20x.png]

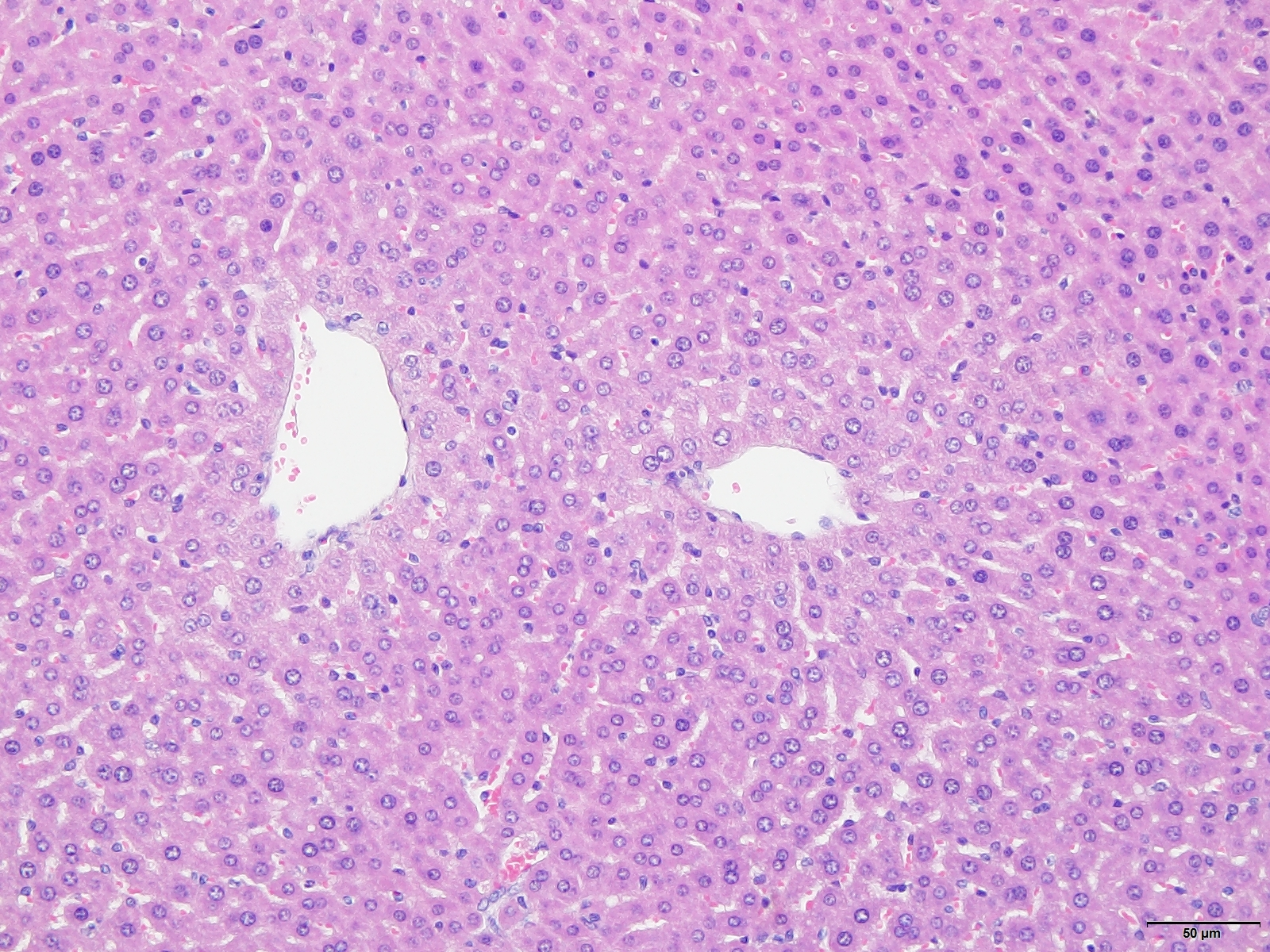

Supplement: Supplementary file 1 [file jox-16-00100-s001.zip › Figure S1/Liver h&E images/2 mg liver 20x.png]

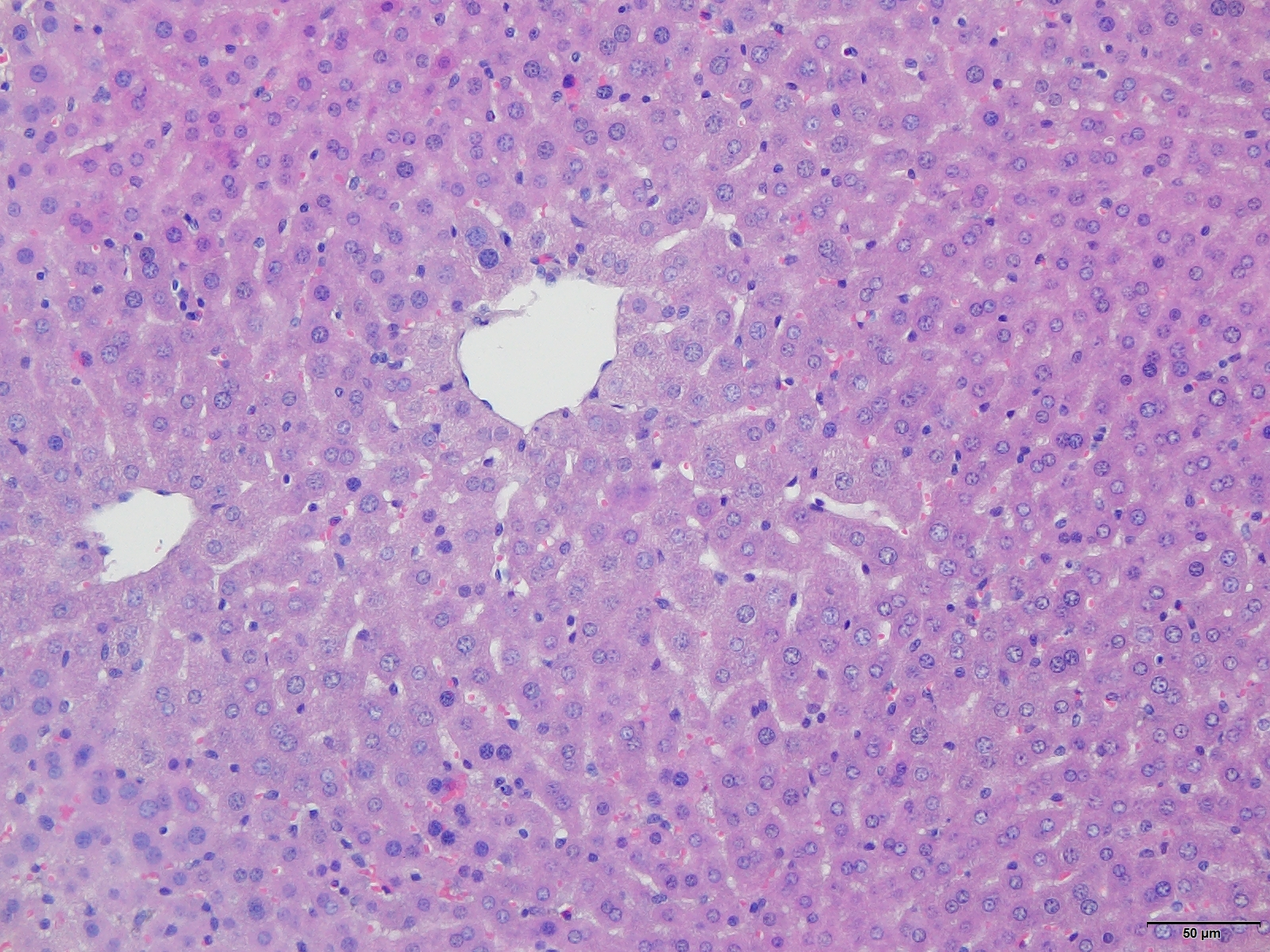

Supplement: Supplementary file 1 [file jox-16-00100-s001.zip › Figure S1/Liver h&E images/control liver 20x.png]

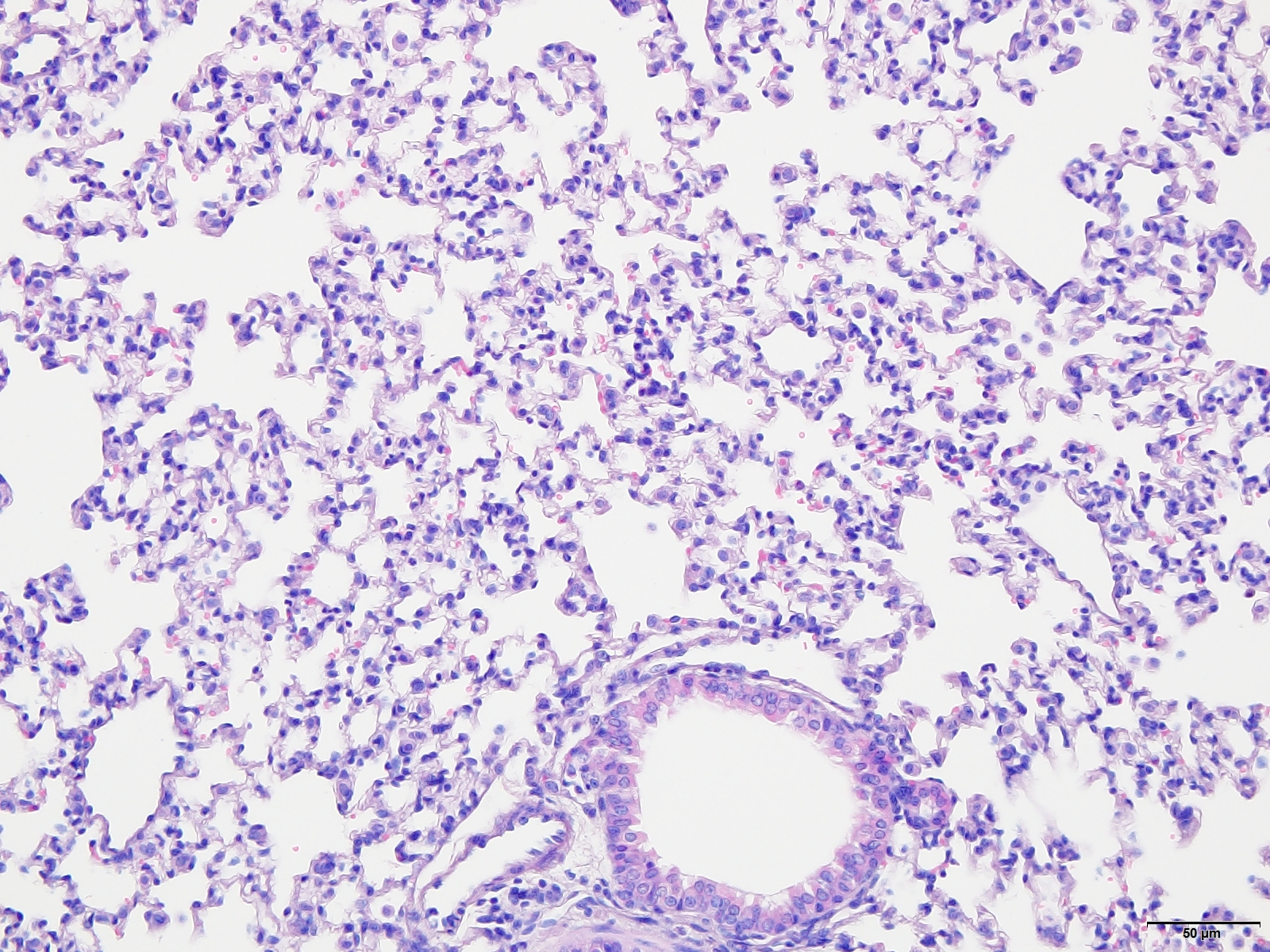

Supplement: Supplementary file 1 [file jox-16-00100-s001.zip › Figure S1/Lungs h&E images/0.5 mg lung 20x.png]

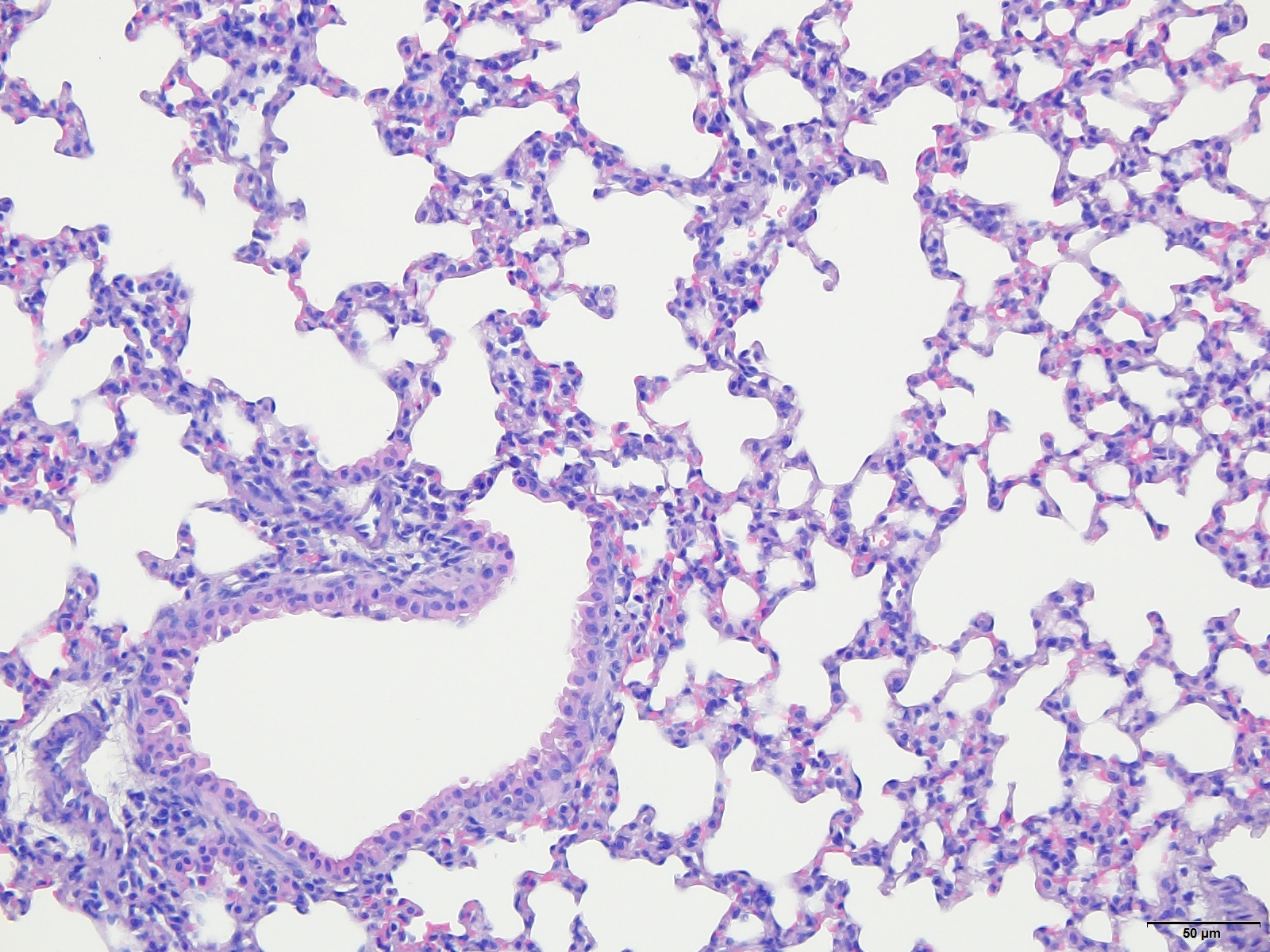

Supplement: Supplementary file 1 [file jox-16-00100-s001.zip › Figure S1/Lungs h&E images/1 mg lung 20x.png]

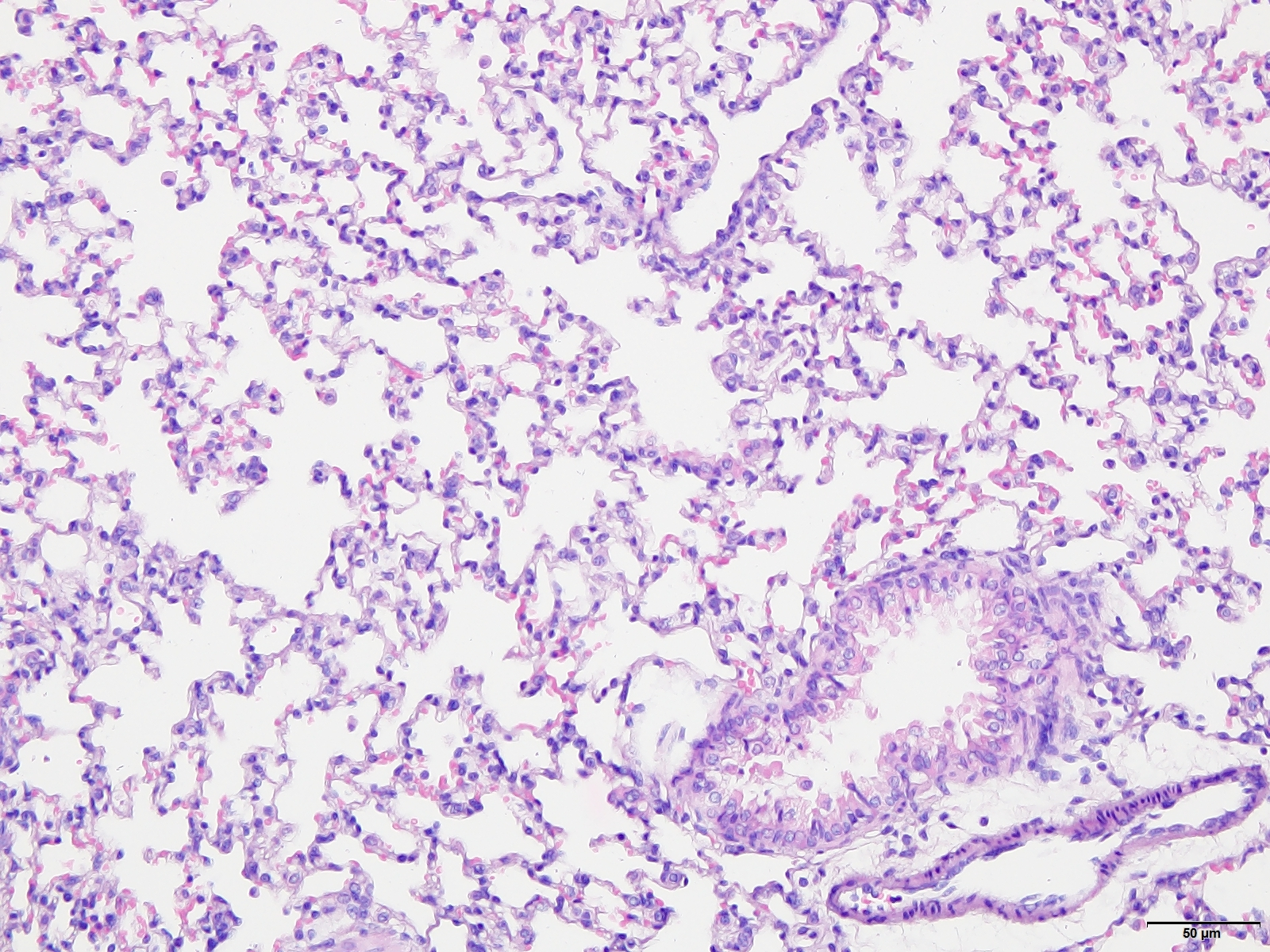

Supplement: Supplementary file 1 [file jox-16-00100-s001.zip › Figure S1/Lungs h&E images/2 mg lung 20x.png]

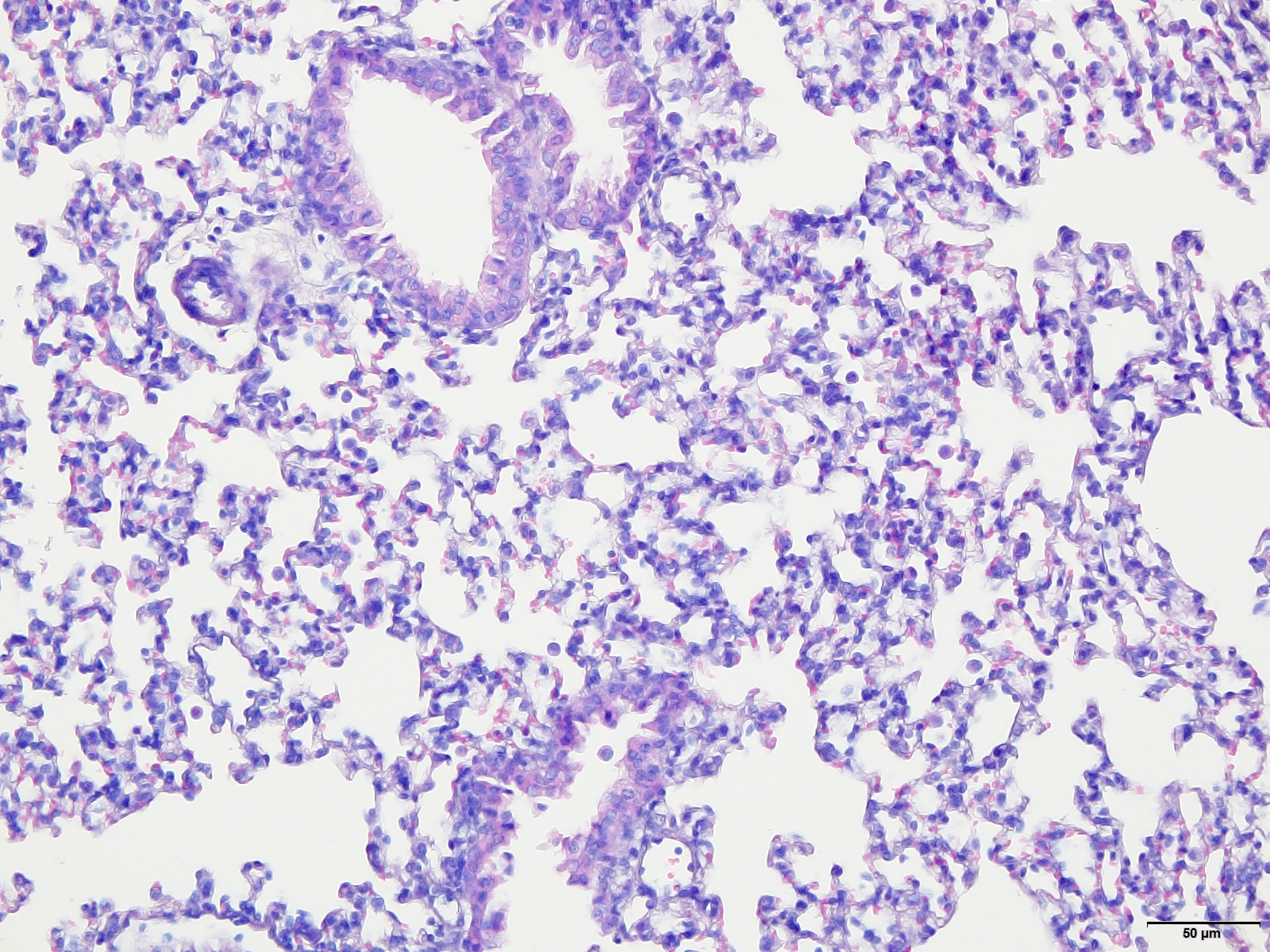

Supplement: Supplementary file 1 [file jox-16-00100-s001.zip › Figure S1/Lungs h&E images/control lung 20x.png]

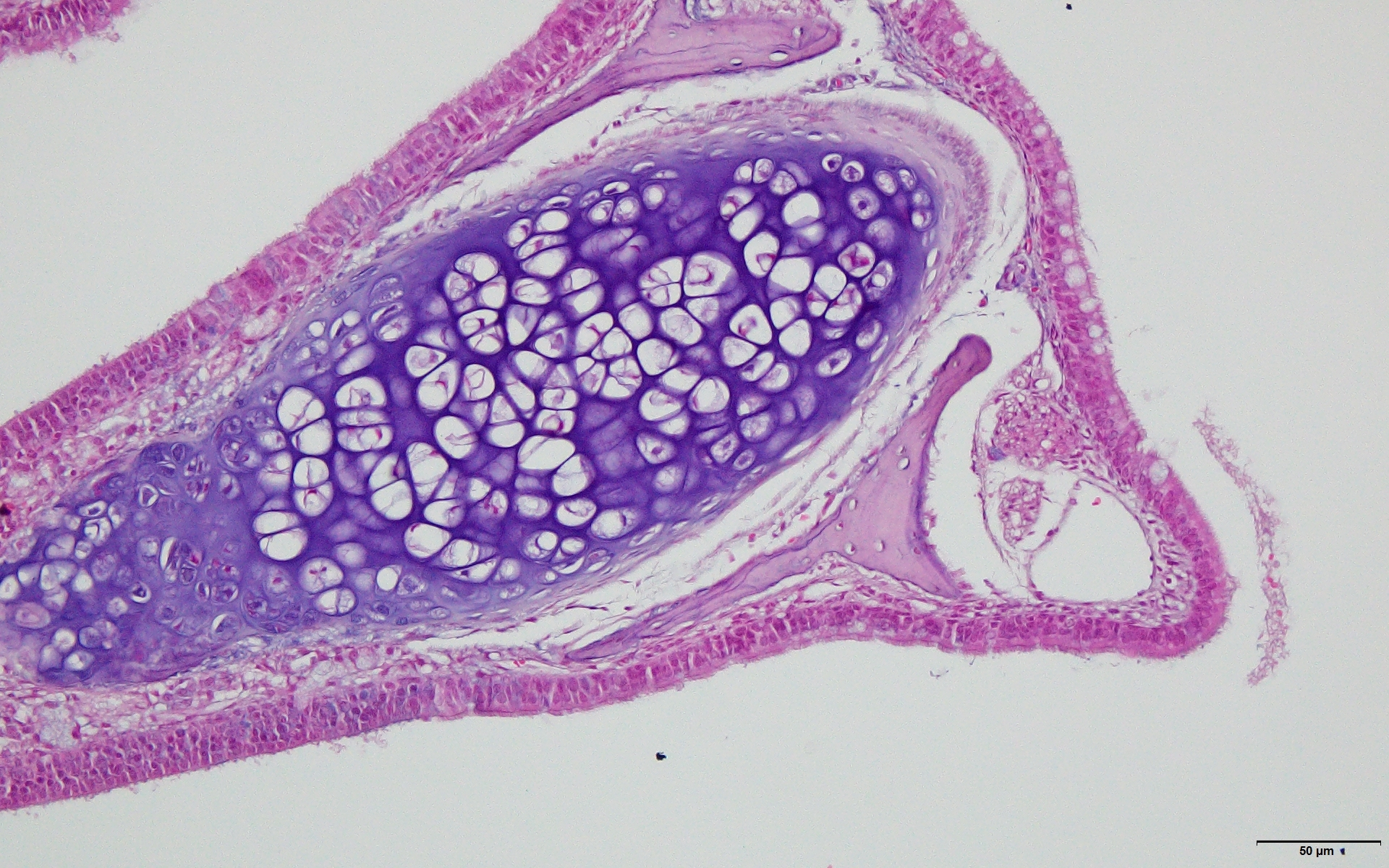

Supplement: Supplementary file 1 [file jox-16-00100-s001.zip › Figure S1/Nasal mucosa h&E images/0.5 mg nasal mucosa 20x.png]

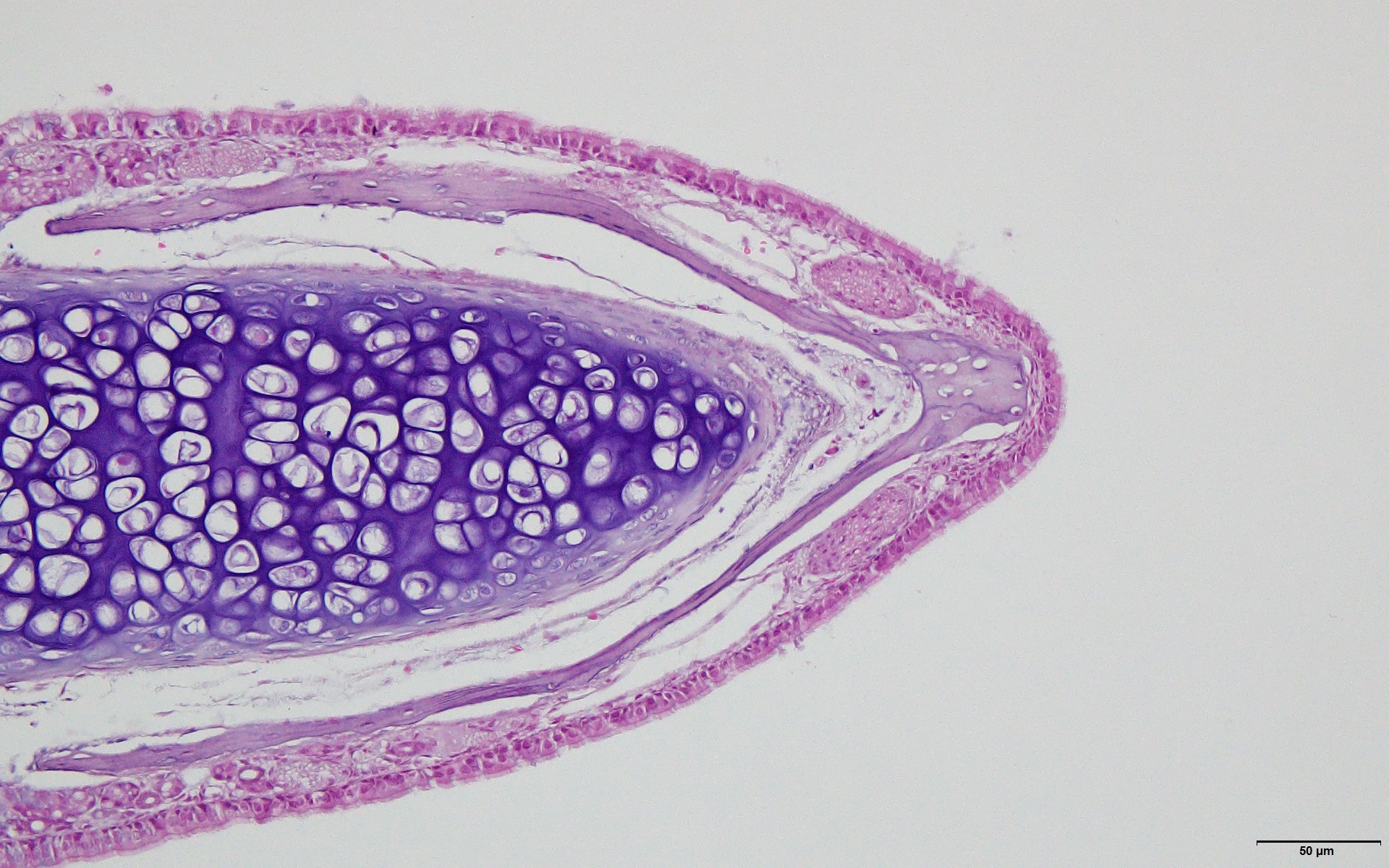

Supplement: Supplementary file 1 [file jox-16-00100-s001.zip › Figure S1/Nasal mucosa h&E images/1 mg nasal mucosa 20x.png]

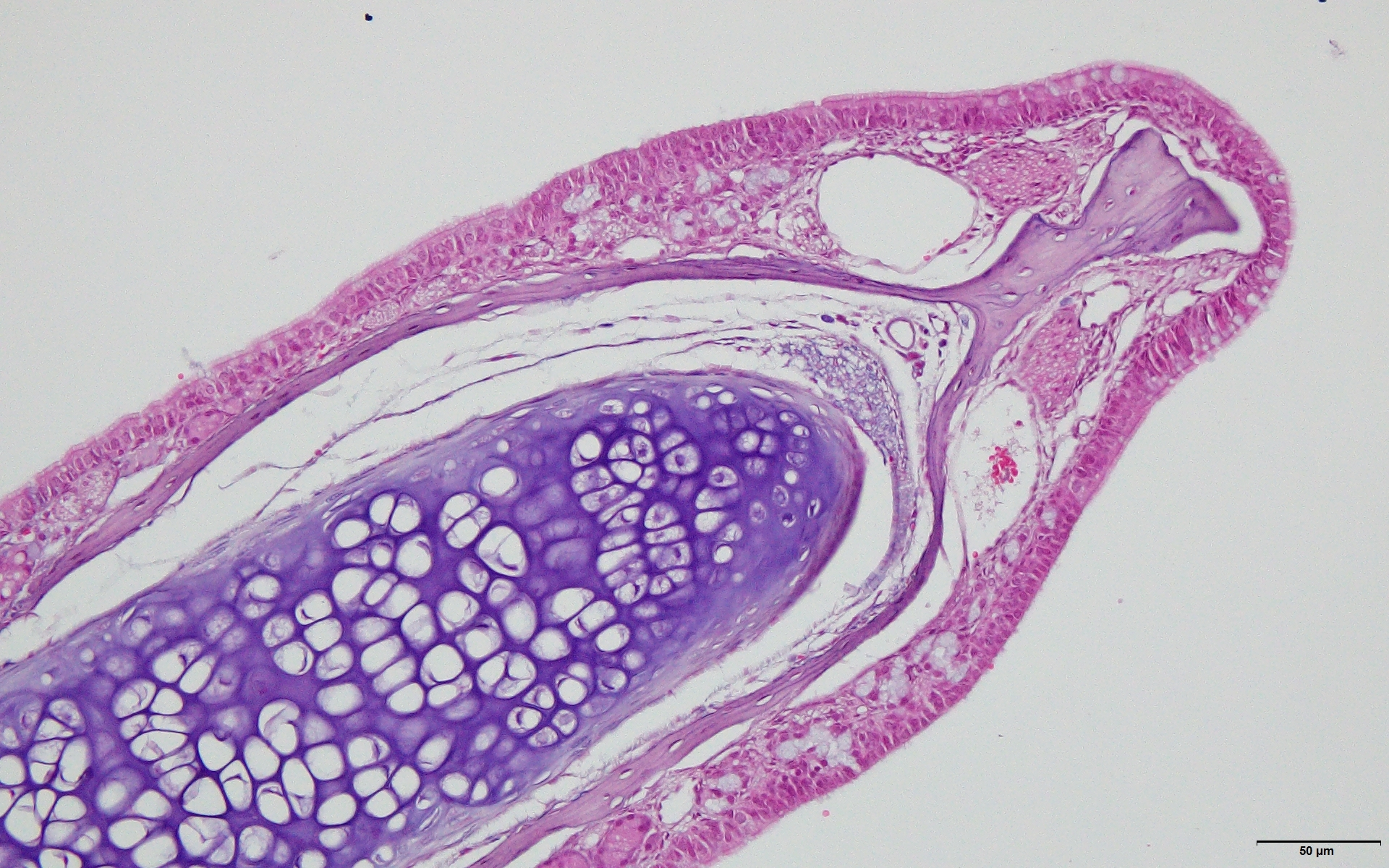

Supplement: Supplementary file 1 [file jox-16-00100-s001.zip › Figure S1/Nasal mucosa h&E images/2 mg nasal mucosa 20x.png]

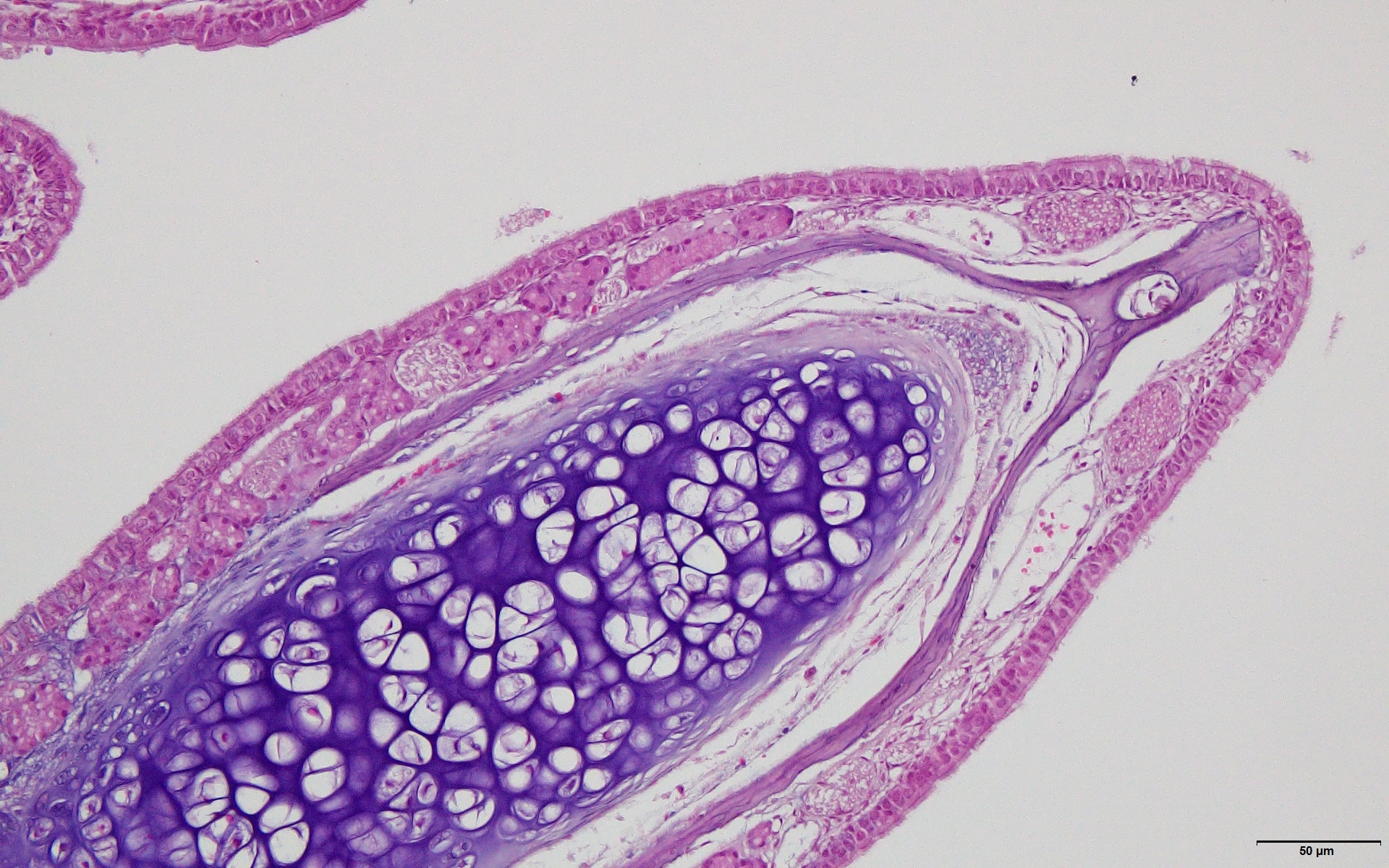

Supplement: Supplementary file 1 [file jox-16-00100-s001.zip › Figure S1/Nasal mucosa h&E images/control nasal mucosa 20x.png]

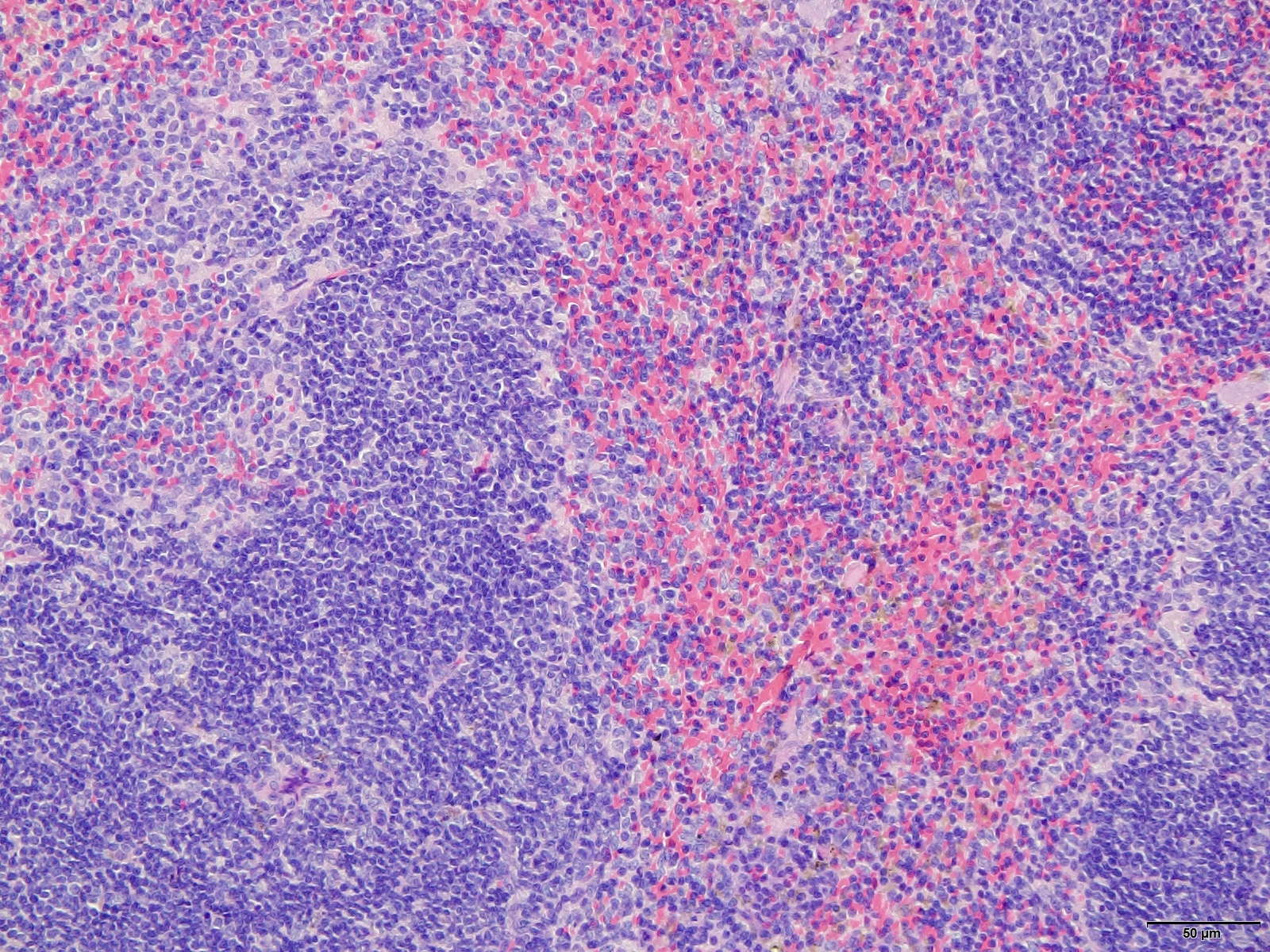

Supplement: Supplementary file 1 [file jox-16-00100-s001.zip › Figure S1/Spleen h&E images/0.5 mg spleen 20x.png]

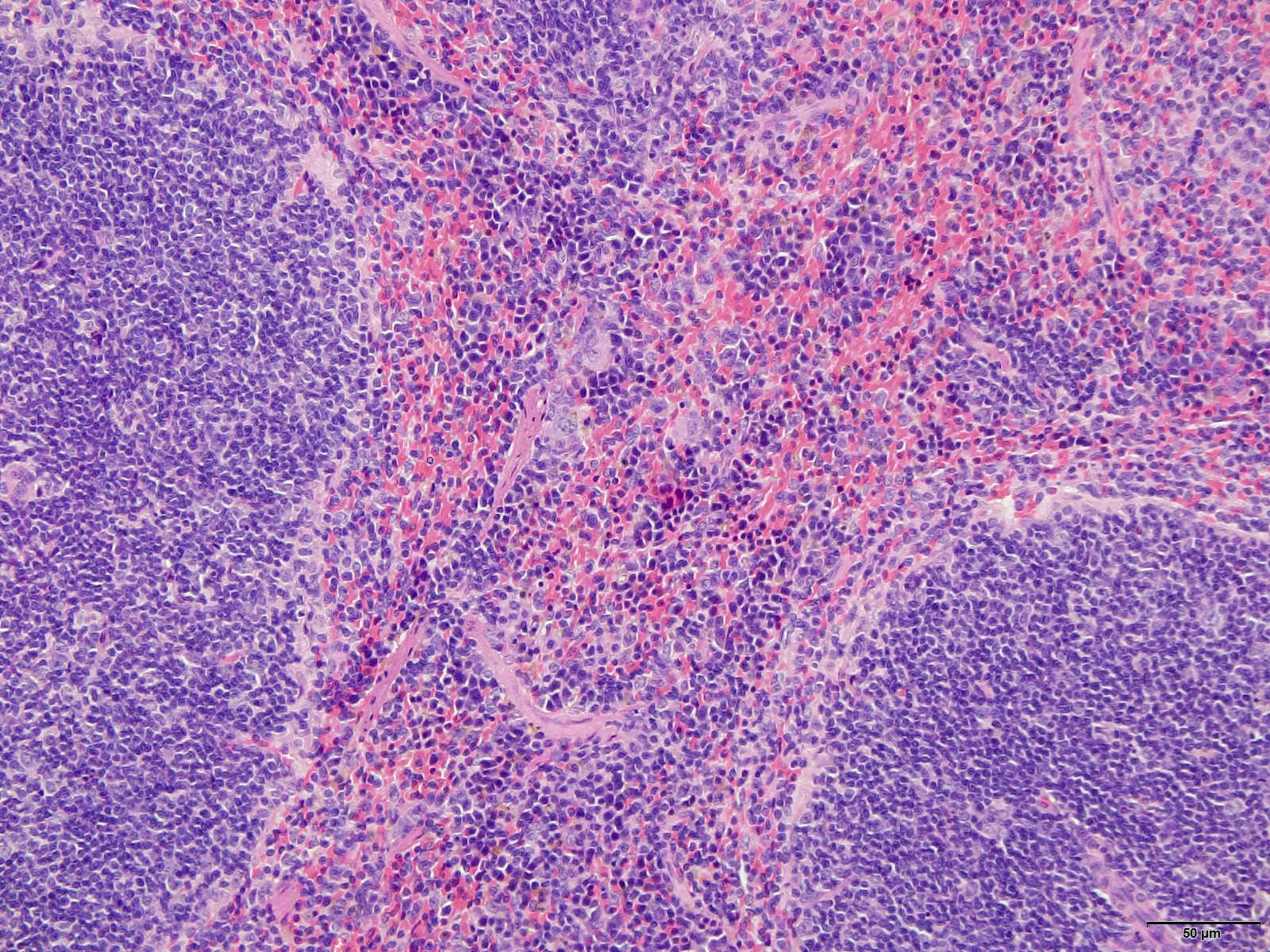

Supplement: Supplementary file 1 [file jox-16-00100-s001.zip › Figure S1/Spleen h&E images/1 mg spleen 20x.png]

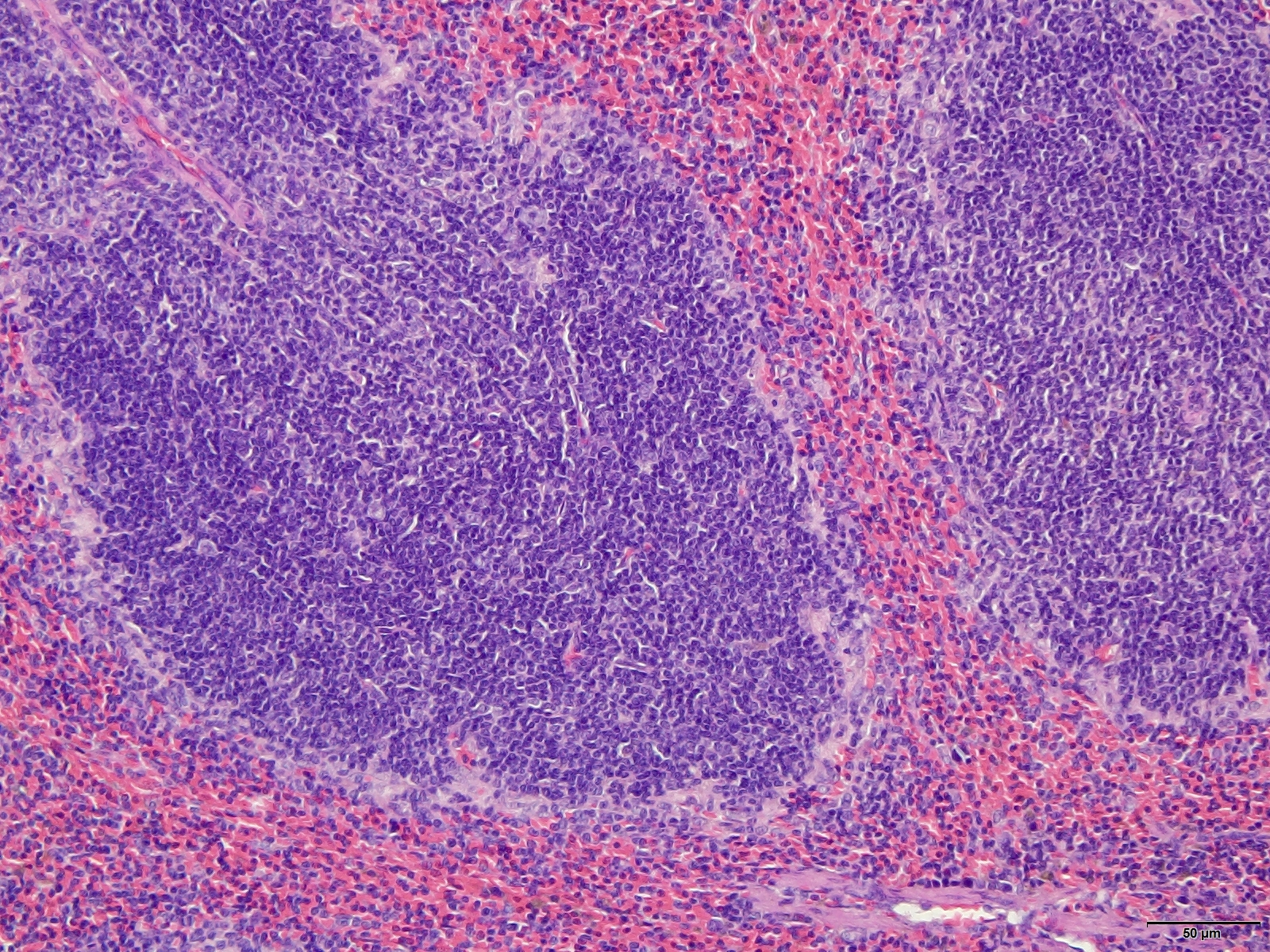

Supplement: Supplementary file 1 [file jox-16-00100-s001.zip › Figure S1/Spleen h&E images/2 mg spleen 20x.png]

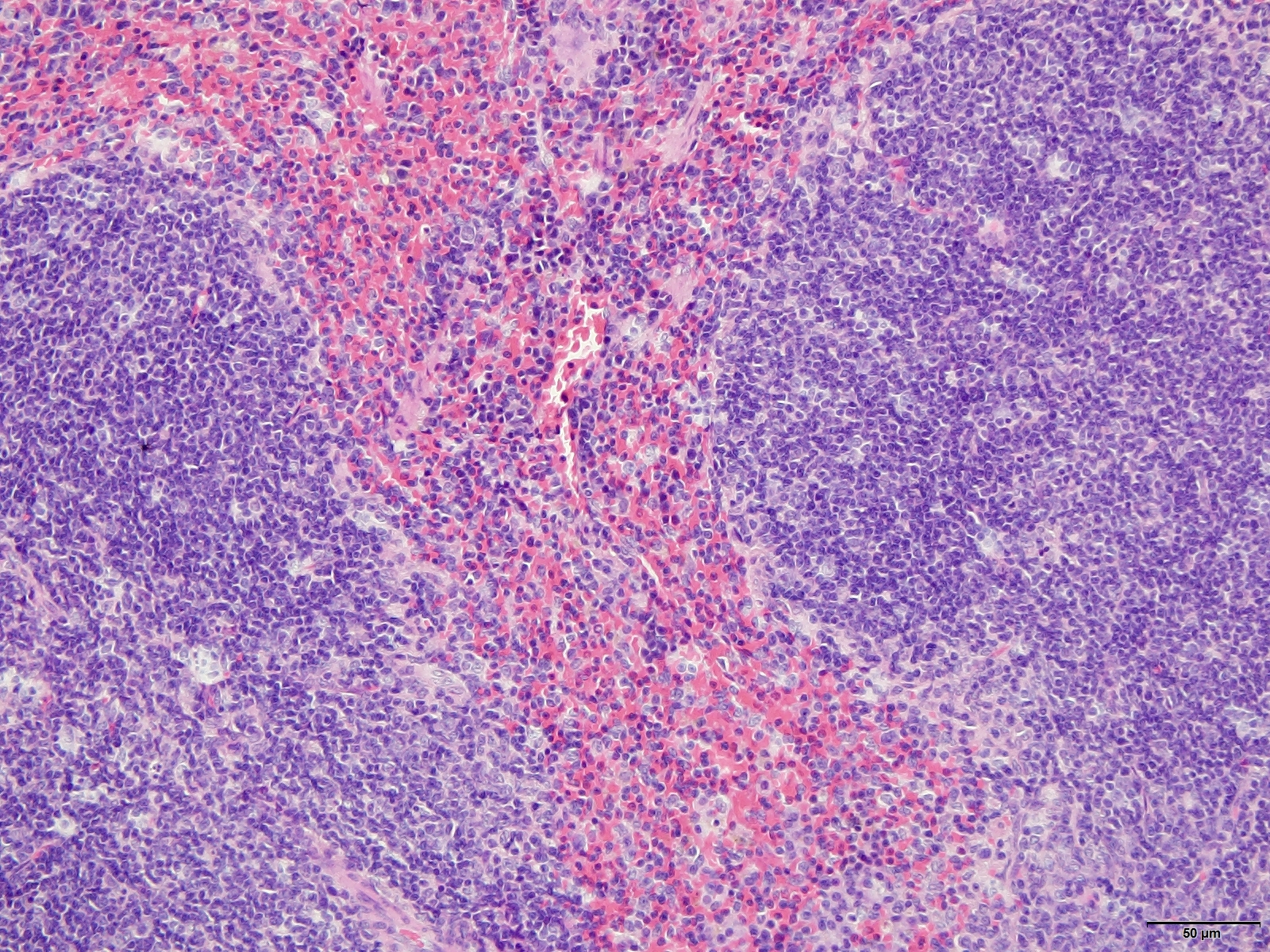

Supplement: Supplementary file 1 [file jox-16-00100-s001.zip › Figure S1/Spleen h&E images/control spleen 20x.png]
